# MEG sensor and source measures of visually induced gamma-band oscillations are highly reliable

**DOI:** 10.1016/j.neuroimage.2016.05.006

**Published:** 2016-08-15

**Authors:** H.-R.M. Tan, J. Gross, P.J. Uhlhaas

**Affiliations:** Centre for Cognitive Neuroimaging (CCNi), Institute of Neuroscience and Psychology (INP), College of Medical, Veterinary and Life Sciences, College of Science and Engineering, University of Glasgow, 58 Hillhead Street, Glasgow G12 8QB, United Kingdom

**Keywords:** Test–retest reliability, Visually induced gamma (30–100 Hz) response, Magnetoencephalography (MEG), DICS beamforming, Time-frequency analysis

## Abstract

High frequency brain oscillations are associated with numerous cognitive and behavioral processes. Non-invasive measurements using electro-/magnetoencephalography (EEG/MEG) have revealed that high frequency neural signals are heritable and manifest changes with age as well as in neuropsychiatric illnesses. Despite the extensive use of EEG/MEG-measured neural oscillations in basic and clinical research, studies demonstrating test–retest reliability of power and frequency measures of neural signals remain scarce. Here, we evaluated the test–retest reliability of visually induced gamma (30–100 Hz) oscillations derived from sensor and source signals acquired over two MEG sessions. The study required participants (N = 13) to detect the randomly occurring stimulus acceleration while viewing a moving concentric grating. Sensor and source MEG measures of gamma-band activity yielded comparably strong reliability (average intraclass correlation, ICC = 0.861). Peak stimulus-induced gamma frequency (53–72 Hz) yielded the highest measures of stability (ICC_sensor_ = 0.940; ICC_source_ = 0.966) followed by spectral signal change (ICC_sensor_ = 0.890; ICC_source_ = 0.893) and peak frequency bandwidth (ICC_sensor_ = 0.856; ICC_source_ = 0.622). Furthermore, source-reconstruction significantly improved signal-to-noise for spectral amplitude of gamma activity compared to sensor estimates. Our assessments highlight that both sensor and source derived estimates of visually induced gamma-band oscillations from MEG signals are characterized by high test–retest reliability, with source derived oscillatory measures conferring an improvement in the stability of peak-frequency estimates. Importantly, our finding of high test–retest reliability supports the feasibility of pharma-MEG studies and longitudinal aging or clinical studies.

## Highlights

• Visually induced gamma activity shows high test–retest reliability.
• Peak gamma frequency is more reliable than its spectral modulation and bandwidth.
• Sensor and source measures of gamma brain activity show comparably high stability.
• Source localization improves signal-to-noise-ratio of measured spectral modulation.
• Range of peak gamma frequency is consistent across early and higher visual cortical areas.

## Introduction

Rhythmic activity is ubiquitous in the cortical brain and manifests a range of frequencies (Buzsaki, 2006, Buzsáki and Draguhn, 2004). These oscillations are remarkably well preserved across species and different frequencies have been associated with distinct cognitive-behavioral states (Buzsáki and Watson, 2012, Buzsáki et al., 2013). In particular, gamma (30–100 Hz) oscillations are thought to play an important role in local and large-scale cortical processing (Bastos et al., 2014, Bosman et al., 2012, Fries, 2009, Roberts et al., 2013), as supported by a range of studies showing a modulation of gamma-band oscillations with cognitive processes such as perception (e.g. Beauchamp et al., 2012, Gross et al., 2007, Wyart and Tallon-Baudry, 2008), attention (e.g. Fries et al., 2001, Ray et al., 2013, Womelsdorf et al., 2006) and memory (e.g. Carr et al., 2012, Colgin et al., 2009, Sederberg et al., 2007).

Given its potential role in routing information within brain networks (Fries, 2015) rhythmic activity at gamma frequencies in response to visual stimuli has received considerable interest in both invasive and non-invasive electrophysiology. Specifically, visually induced brain responses at gamma frequencies have been shown to vary with stimulus properties (Jia et al., 2013, Perry et al., 2013; see also Box 1 in Tan et al., 2013) such as contrast (Ray and Maunsell, 2010), stimulus type (Hermes et al., 2014, Hermes et al., 2015), stimulus velocity (e.g. Friedman-Hill et al., 2000, Gray et al., 1990, Lima et al., 2011, Muthukumaraswamy, 2013), and the temporal expectation of reward (Lima et al., 2011). Moreover, the peak frequency of visually induced gamma brain oscillations shows high heritability in monozygotic twins (van Pelt et al., 2012), with concordances comparatively lower for heterozygotic twins and lowest between non-related individuals.

In addition to their role during normal brain functioning, visually-induced oscillations have been investigated in several neuropsychiatric disorders, such as schizophrenia and autism spectrum disorders, as a means of deriving insights into the underlying circuit dysfunctions (e.g. Sun et al., 2013, Tan et al., 2013). Collective experimental and theoretical studies provide evidence that cortical gamma-band activity is predominantly generated through rhythmic synaptic inhibition, which temporally coordinates windows of excitability in principal cells (Bartos et al., 2007, Buzsáki and Wang, 2012, Wang, 2010, Whittington et al., 2000). These recurring frames of excitability provide an efficient and elegant means of organizing and coordinating functional cell ensembles for neural communication (Akam and Kullmann, 2014, Buzsáki and Watson, 2012, Buzsáki, 2010). Along with mutually connected (GABA-mediated) inhibitory neurons (Traub et al., 1996, Wang and Buzsáki, 1996), networks of recurrent (AMPA-mediated) excitatory-inhibitory neurons also contribute to the genesis of gamma oscillations (Brunel and Wang, 2003, Tiesinga and Sejnowski, 2009, Wang, 2010). Importantly, changes in cellular parameters have been found to affect the coordination of excitatory and inhibitory processes (Bernard et al., 2000, Hashimoto et al., 2008, Lewis et al., 2005) and may lead to impairments in generating high-frequency oscillations (Ben-Ari et al., 2004, Ben-Ari et al., 2012, Gonzalez-Burgos and Lewis, 2008, Schnitzler and Gross, 2005, Uhlhaas and Singer, 2012).

Despite prevalent use of visually-induced gamma-band responses during normal brain functioning and in neuropsychiatric populations, evidence for the reliability of EEG/MEG-derived oscillatory measures remains scarce. However, for measures of high-frequency neural activity to be useful neurophysiological “spectral fingerprints” (Siegel et al., 2012) and potentially serve as biomarkers or endophenotypes in clinical research, it is essential that these parameters are highly reliable and robust. To the best of our knowledge, there are only two studies that systematically assessed the test–retest reliability of visually induced neural oscillations from EEG/MEG signals. The study by Muthukumaraswamy et al. (2010) revealed high intraclass correlation (ICC) values (0.8–0.98) for spectral measures of visually elicited gamma (40–60 Hz) band responses to static visual gratings across multiple assessments for source-derived MEG-signals. Specifically, they found highest repeatability for peak frequency, followed by its corresponding spectral signal change and bandwidth. In addition, Fründ et al. (2007) observed that the magnitude and frequency of participants' EEG responses to large (vs. small) foveally presented static visual gratings were strongly (Pearson's) correlated between sessions, highlighting the dependency of visually induced gamma activity on stimulus properties.

To further examine the test–retest reliability of visually-induced responses in neuromagnetic data, we employed a modified moving visual stimulus protocol (van Pelt et al., 2012) developed by Hoogenboom et al. (2006) that has been shown to yield gamma-band responses with robust signal-to-noise-ratio (SNR). Specifically, the current study assessed estimates of peak frequency, spectral modulation and spectral bandwidth across measurements to comprehensively assess the reliability of visually-induced responses from both sensor and source derived MEG signals. Additionally, we evaluated the SNR for sensor vs. source estimates of visually-induced high-frequency activity, which is a question relevant to both basic and applied MEG research.

## Methods

The University of Glasgow College of Science and Engineering Ethics Committee approved the experimental protocol, in which the present experiment was part of a battery of sensory processing tasks performed during each MEG session.

### Participants

Fourteen healthy participants (4 Females; mean age (± SD) = 25 (± 4) years) took part in our study to assess the test–retest reliability of visually induced high frequency neural oscillations over two MEG sessions (range 1–11 days; mean (± SD) = 4 (± 3) days apart). Participants were recruited from the University of Glasgow School of Psychology participant pool, provided informed consent prior to the experiment and were compensated (at the standard rate of £6/h) for their time. All participants were right handed (Edinburgh Handedness Test; Oldfield, 1971), characterized by normal or corrected vision and had no known neurological disorders.

Prior to each MEG session, scheduled at the same time of the day, each participant filled in a brief questionnaire which assessed differences in caffeine intake, smoking habits, alcohol consumption, and hours of sleep as well as general well-being prior to each measurement. As previously reported (Tan et al., 2015) participants' responses did not differ across sessions. Additionally, female participants took part in the study within the first 5–10 days during the follicular phase of their menstrual cycle in both MEG sessions to control for potential influence of hormonal fluctuations (Epperson et al., 2002).

### Stimuli and task

We employed a foveally presented moving visual grating stimulus (Supplementary SFig. 1) that has been observed to induce robust MEG gamma band response in the human visual cortex (Hoogenboom et al., 2006). The visual grating was presented at a viewing distance of 186 cm in front of the seated participants. Each trial began with the presentation of a central fixation spot (Gaussian diameter: 0.5°) for 500 ms. The contrast of the fixation spot is subsequently reduced by 40% for 1500 ms, indicating the upcoming presentation of the moving circular sine wave gratings. The ensuing concentric moving grating (2.7 cycles/°; contrast: 100%; 5° visual angle; velocity: 0.75°/s) contracted towards the fixation spot and accelerated (velocity: 1.2°/s) randomly between 750–3000 ms post grating presentation onset. Participants were tasked to indicate the detection of this acceleration with a button press within 700 ms of its occurrence. Each trial lasted for ~ 4–6 s long and during the inter-trial interval (1000 ms) participants were provided with feedback as to whether the speed of their response was adequate, too fast or too slow. Rare incidences (10%) in which no acceleration occurred were interspersed within a sequence of 80 trials that made up a block of the visual task. We provided performance accuracy feedback during the short break after each of the 3 task blocks.

### Neuroimaging acquisition

MEG data were acquired using a 248-channel magnetometer system (MAGNES® 3600 WH, 4D-Neuroimaging, San Diego) while participants engaged in the task, sitting upright within an electromagnetically shielded room. For each participant, a suitable MEG seat position was determined and marked during the first session. Every attempt was taken to keep this seat position and the MEG system's helmet (housing the SQUID sensors) in the same configuration prior to each acquisition so as to minimize the variance of participants' head and sensors' positioning across runs and sessions. Head position stability was assessed before and after each acquisition run via five indicator coils attached relative to the (left, right preauricular and nasion) fiducials, and were co-digitized with participants' head-shape (FASTRAK®, Polhemus Inc., VT, USA) for subsequent co-registration with individual MRI (1 mm^3^ T1-weighted; 3D MPRAGE). The MEG, touch-pad response (LUMItouch™, Photon Control Inc., BC, Canada) and eye-tracker (EyeLink 1000; SR Research Ltd., Ontario, Canada) signals were sampled synchronously at 1017.25 Hz, with online 0.1 Hz high-pass filtering.

### MEG data processing

All data processing and analyses were performed using Fieldtrip Toolbox functions (http://fieldtrip.fcdonders.nl; Oostenveld et al., 2011) and additional scripts developed within MATLAB® (The MathWorks, Natick, MA). Faulty sensors (mean (± SEM) = 10 ± 2 per session, visually identified) with large signal variance or whose signals were flat were removed and interpolated using nearest-neighbor averaging procedure. One MEG-measurement was corrupted by global noise and technical issues during one of the two acquisition sessions. Accordingly, this participant was excluded from the analyses reported here (i.e. N = 13).

Raw MEG signals from correctly responded trials were epoched from − 1500 to + 2000 ms relative to grating stimulus onset (0 ms), with linear trends removed, power-line (50 Hz) notch-filtered, and ‘de-noised’ relative to reference MEG channel signals. Raw trials were visually inspected and trials with obvious artifacts (muscle, squid jumps etc.) were excluded. Subsequently, Independent Component Analysis was used to isolate and to reject ocular-movement and cardiac components from the MEG signals, yielding on average (± SEM) 180 (± 7) artifact-free trials for each participant and session.

### Time-frequency analysis on sensor and source signals

**Fig. 1:**
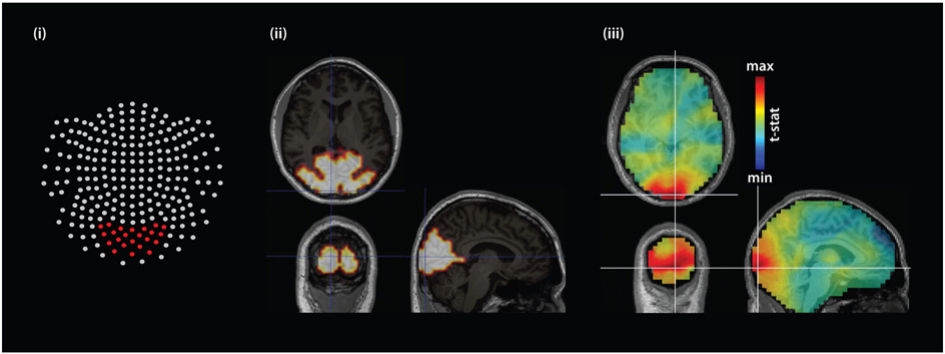
Description of sensor and source space from which neuromagnetic signals were derived. (i) Two-dimensional sensor array layout of the 4D Neuroimaging (San Diego, USA) MEG system, with visual sensors highlighted in red. (ii) Sources delineated within primary visual brain regions of interest shown in axial, coronal and sagittal views. (iii) Stimulus vs. baseline source statistics of one participant (S09, session 2) interpolated over participant's MNI-normalized brain shown in axial, coronal and sagittal views. The white cross-marks highlight the maximally activated voxel (based on FDR-corrected non-parametric T-stats value; p < 0.05). Refer to Methods, Results, and Table 3 for further details.

For sensor level analysis, artifact-free neuromagnetic time series were transformed to planar gradient signals (Bastiaansen and Knosche, 2000) prior to time-frequency analyses and subsequently recombined. Similar to previous work (e.g. Hoogenboom et al., 2006, van Pelt et al., 2012) we focused our sensor-level analysis on the spectral power time-series derived from the 23 parieto-occipital sensors (‘A135’, ‘A136’, ‘A137’, ‘A138’, ‘A139’, ‘A162’, ‘A163’, ‘A164’, ‘A165’, ‘A166’, ‘A167’, ‘A184’, ‘A185’, ‘A186’, ‘A187’, ‘A188’, ‘A202’, ‘A203’, ‘A204’, ‘A205’, ‘A219’, ‘A220’, ‘A221’) over visual cortex (Fig. 1_(i)_; Supplementary SFig. 3).

At the source level, prior work (Hoogenboom et al., 2006, Muthukumaraswamy et al., 2010) and preliminary assessment of source-level data indicated that strongest signals were generated within the calcarine, visual lingual, and occipital areas. Given that prior findings have consistently reported visually-induced cortical sources significantly associated with the high frequency oscillations, average signals from visual cortical regions (bilateral calcarine, cuneus, lingual, superior, mid and inferior occipital cortical areas) were initially derived employing the Anatomical Automatic Labeling atlas (ROI_MNI_V4.nii; Tzourio-Mazoyer et al., 2002) implemented within SPM/fieldtrip (see Fig. 1_(ii)_). However, this led to a loss of signal power, which suggested source-specificity in the stimulus-induced modulation (Supplementary SFig. 3_(iii)_, see also SFigs. 5–7). We therefore determined the maximally induced voxel for each individual for each MEG session.

**Fig. 2:**
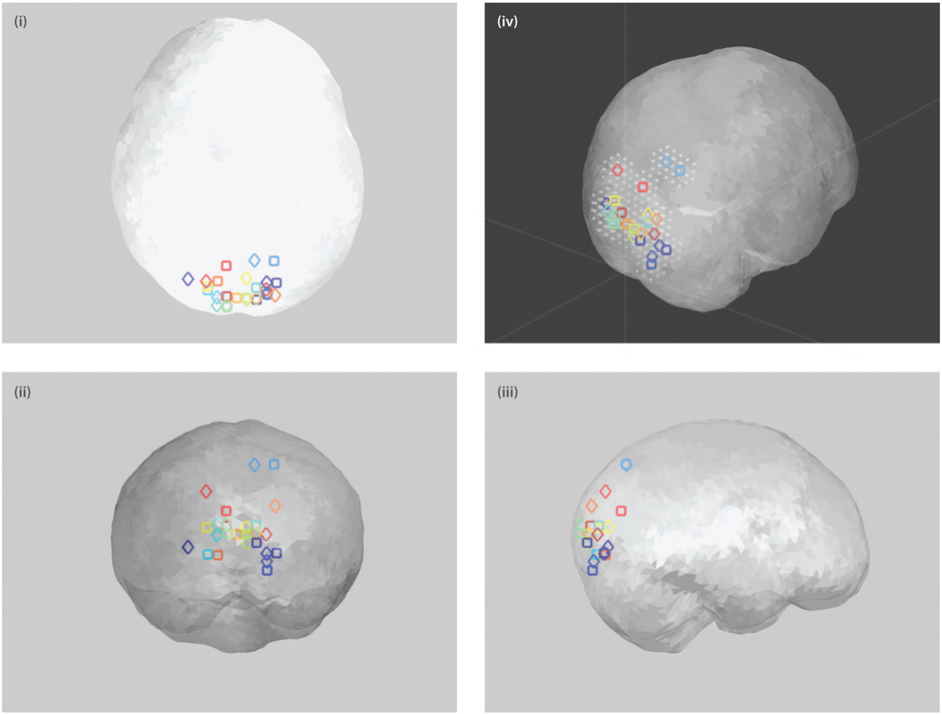
Summary of all participants' maximally modulated voxels in response to moving visual grating stimulus for both sessions. Maximally modulated voxels shown in (i) axial, (ii) coronal and (iii) sagittal views within ‘glass’ brain volume. Each participant's maximally modulated voxel is color-coded as in Figs. 4–6 with square and diamond markers denoting non-parametric T-stats derived maximum in sessions 1 and 2, respectively. (iv) 3-D brain volume view of maximally modulated voxels surrounded by their neighboring 26 voxels whose signals were incorporated in the time-frequency analyses. Refer to Methods for further details.

To this end, a 30–100 Hz broadband time-frequency decomposition (frequency centered at 65 Hz; ± 35 Hz taper smoothing; 10 ms temporal resolution; 50% overlapping with 500 ms time window) was performed on the artifact-free epochs prior to the derivation of common source spatial filters using the DICS inverse-solution algorithm (Gross et al., 2001). Subsequently, bootstrap resampling source statistics was performed (with 1000 Monte Carlo repetitions) between stimulus duration of interest (StimDur) and baseline (500–2000 ms and − 1500–0 ms relative to moving grating onset, respectively) to determine individual participants' maximal source statistic (see Fig. 1_(iii)_; Supplementary SFig. 2). For each participant and session, virtual sensors' signals were extracted from the maximally modulated source (FDR-corrected, alpha = 0.05) as well as its corresponding 26 surrounding voxels using individual MNI-normalized source model grid (6 mm resolution). Fig. 2 (and Supplementary SFig. 2) provides a summary of all participants' maximally modulated source location for both sessions.

### Derivation of oscillatory parameters and reliability analysis

**Fig. 3:**
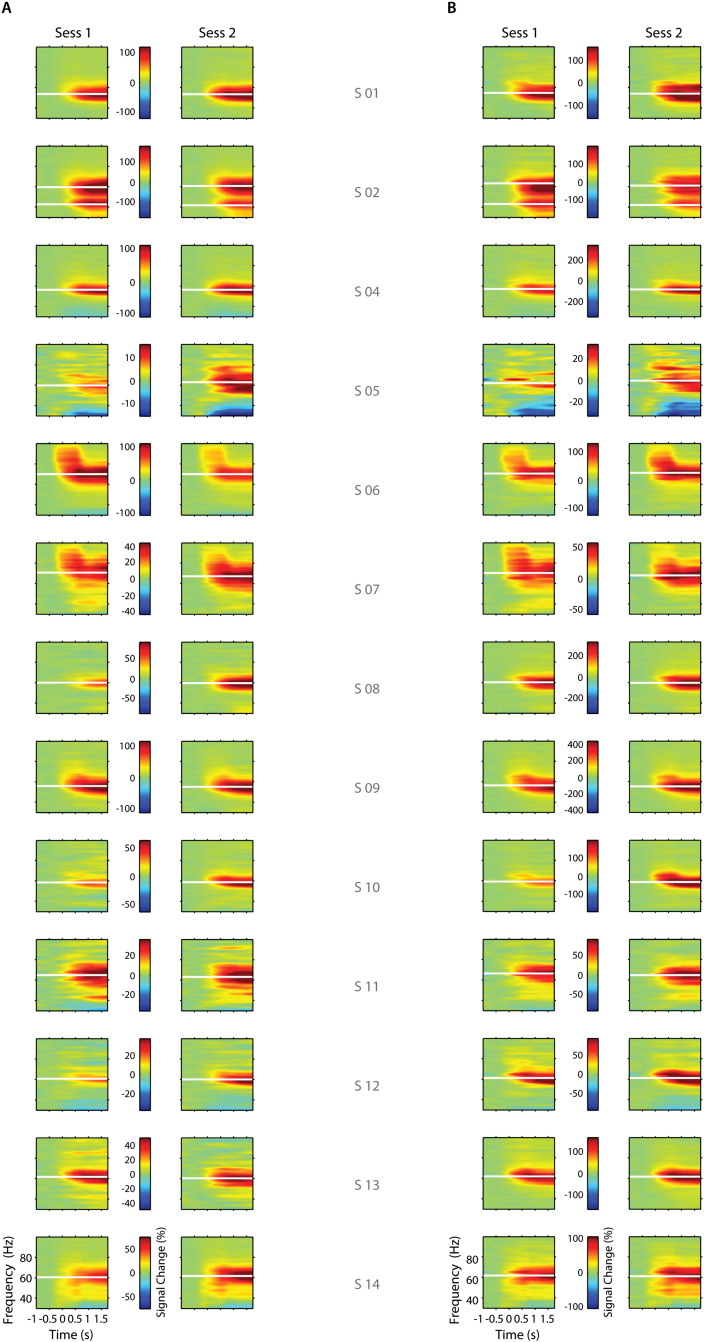
Individual time-frequency plots. Time-frequency plots for signal change for sensor (A) and source (B) derived signals in response to the moving visual stimulus for both MEG sessions. Horizontal white lines denote peak frequency derived from Gaussian fits (see Supplementary SFig. 4). Participant S02 exhibited double peaks.

For both sensor and source derived signals multi-taper fast-fourier time-frequency decomposition (± 2 Hz taper smoothing and 10 ms temporal resolution; 2 Hz resolution from 30 Hz to 100 Hz) was performed with 50% overlapping 500 ms time window on the artifact-free epochs. All spectral power time-series were expressed as relative change to baseline (from − 1500 ms to 0 ms prior to visual grating stimulus onset; Supplementary SFig. 4; Fig. 3). Induced sustained spectral modulations were averaged over the period of 500 ms–2000 ms post grating stimulus onset (see Supplementary SFig. 4; SFig. 3) for each frequency interval within the 30–100 Hz range; avoiding transient visual response onset and any preparatory behavioral responses.

A 1st order Gaussian fit was performed on these time-averaged spectral time-series (Campbell et al., 2014, Haegens et al., 2014) to determine (a) peak response frequency, and the corresponding (b) signal change modulation and (c) bandwidth (i.e. by deriving the full-width-at-half-maximum; FWHM) at this peak frequency (Supplementary SFig. 4). As in previous research (e.g. Hoogenboom et al., 2006), in cases where a participant manifested double gamma-band peaks (e.g. S02), the higher gamma-band peak was selected for subsequent analysis.

Adopting a similar approach to previous reliability assessments (Muthukumaraswamy et al., 2010, Tan et al., 2015) we calculated the intraclass correlation (ICC; Shrout and Fleiss, 1979) using Matlab Central file-exchange *ICC.m* function (A. Salarian 2008; implemented with statistical testing based on McGraw and Wong, 1996a, McGraw and Wong, 1996b) to assess the degree of consistency of these spectral variables. Defined as the ratio of between-subject variance and the total variance, ICC assesses the reliability of the repeated measures of an individual's oscillatory parameters by comparing the between-measures variability of each individual to the total variation across all measures and participants. An ICC value of 1 indicates perfect within subject reliability of neural oscillatory measures derived on differing occasions from the same participants, while ICC of 0 indicates no reliability. ICCs were assessed for both sensor and source-derived neuromagnetic parameters. The distributions of parameters of interest were similar across sessions (i.e. insignificant 2-sample Kolmogorov–Smirnov tests), although those of signal change at sensor level and spectral bandwidth at source level from session 1 were marginally skewed (as determined by Lilliefors test of normality). For appropriate application, these, together with their corresponding distributions from session 2, were square root transformed prior to ICC assessments.

### Distributions of peak frequency and signal change within visual cortical regions

The derivation of oscillatory parameters within each participant's set of 12 AAL-parceled (calcarine, cuneus, lingual and occipital gyri; ~ 800 voxels including both hemispheres) visual cortical regions were repeated to further assess the distribution of peak frequency and corresponding signal change within visual cortical regions for each session.

## Results

Participants demonstrated high response accuracy; mean (± SD): 88 (± 5) % and 93 (± 4) % for sessions 1 and 2, respectively. On average (± SD), their response times were 554 ± 71 ms and 536 ± 60 ms, for sessions 1 and 2, respectively. While response accuracy improved on the second session (t_12_ = − 4.451; p < 0.001), mean response times did not differ between sessions (t_12_ = 1.417; p = 0.182).

Maximally modulated visual voxels from all participants were distributed predominantly within the primary visual lingual, calcarine, and occipital areas (Fig. 2). Table 1 lists the SPM coordinates and anatomical labels of each participant's maximum voxels for both sessions. The locations of participants' maximally modulated voxels are not identical between sessions, but are mostly clustered within neighboring voxels (6 mm resolution voxels). The mean (± SD) intra-participant spatial variability of maximally modulated voxel location in our participant dataset is 21 (± 15) mm.

Spectral changes during stimulus presentation for both sessions are shown in Fig. 3 for sensor (A) and source (B) derived MEG signals for all participants. For most participants, the induced gamma frequency response is sustained from about 350 ms post moving grating stimulus onset until end of stimulus presentation and confined to a frequency range of 50–80 Hz. The induced visual gamma responses for both sessions from either sensor or source derived signals show good resemblance. Spectral responses derived from maximally modulated visual ROIs were generally much stronger, reaching a maximal of ~ 450% signal change compared to sensor estimates (~ 200%). For participants with stronger spectral change relative to baseline (> 40%) at the sensor level, the source-derived frequency response revealed stronger modulations (100–400%). Participants for whom spectral change was weak at sensor level (< 25%), the induced spectral modulations were similarly recovered at source level.

**Fig. 4:**
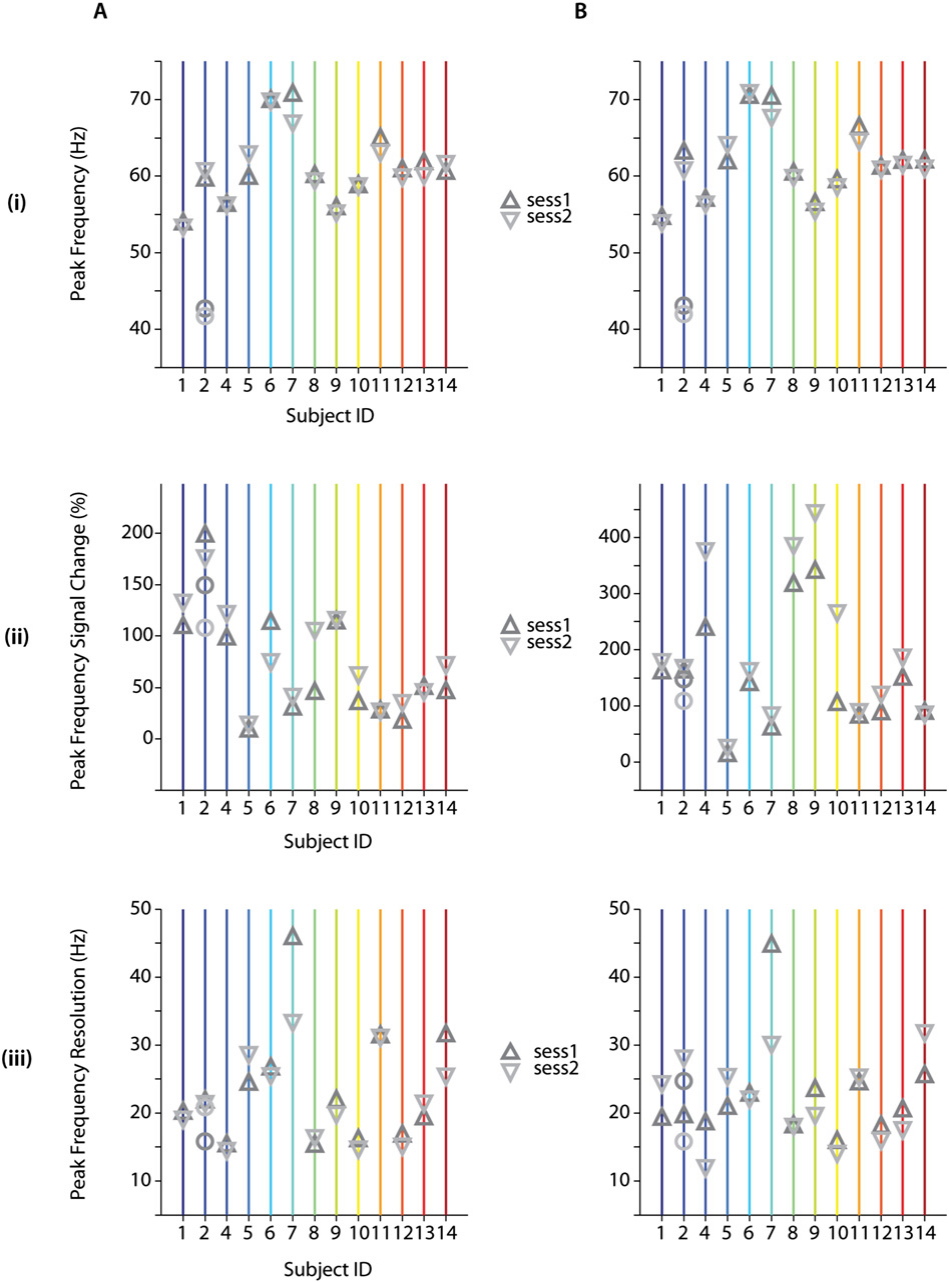
Individually derived visually induced response parameters of interest. Individual values for (i) peak frequency, corresponding (ii) signal change and (iii) frequency bandwidth are extracted for each MEG session at sensor (A) and source (B) space. The second peak frequency of participant with observed double peaks (S02) at sensor level is depicted as circles (i) for both MEG sessions.

For a quantitative measure of repeatability of visually induced gamma response, we assessed the retest reliability of peak frequency, the corresponding spectral signal change and response frequency bandwidth, as determined by the full-width-at-half-maximum (FWHM) of the 1st order Gaussian fit (Supplementary SFig. 4; see Methods). These spectral parameters are summarized in Table 2 and Fig. 4 for both MEG sessions and their respective grand-average time-frequency plots for each session (A, B) are shown in Supplementary SFig. 3. Individual peak frequencies ranged approximately 53–72 Hz for both sensor and source derived spectral measures (Fig. 4A_(i)_, B_(i)_) with an average (± SD) of ~ 61 (± 5) Hz. The signal change ranged between 8–200% (mean (± SD): 74 (± 51) %) and 12–450% (mean (± SD): 175 (± 114) %) for sensor and source derived peak frequencies (Supplementary SFig. 4A_(ii)_, B_(ii)_), respectively. The bandwidth of the peak frequency spanned the range between 12 and 46 Hz for sensor and source derived peak frequencies (Fig. 4A_(iii)_, B_(iii)_) were 23 Hz (± 7) Hz on average (± SD).

**Fig. 5:**
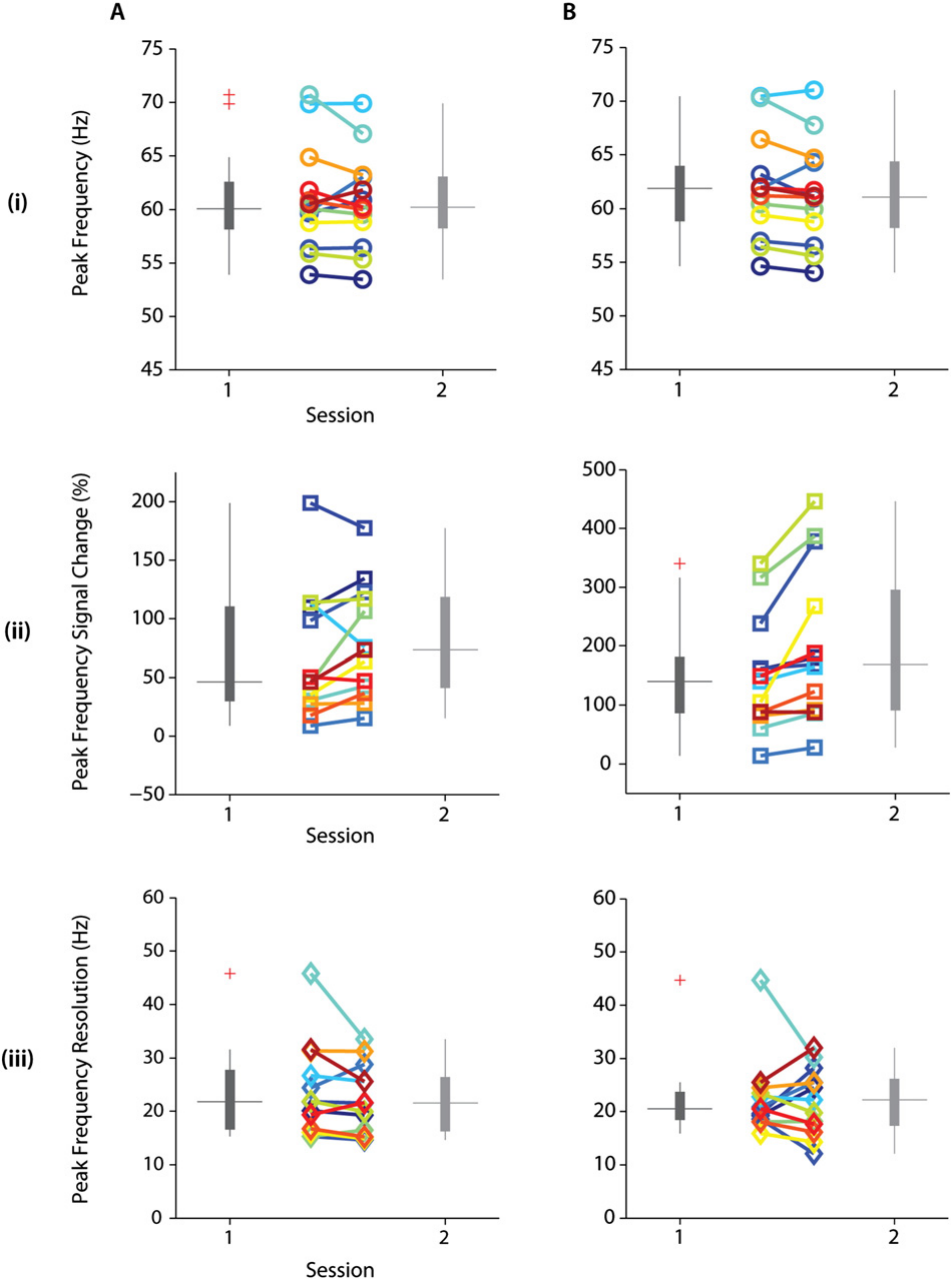
Summary of individually derived visually induced response parameters. Boxplot summary of individually derived parameters of interest (i–iii) are shown for sensor (A) and source (B) derived signals for both MEG sessions. Individual values for each parameter are similarly color-coded by participants (e.g. Fig. 4A, B).

Sensor as well as source-derived peak frequency and spectral resolution did not differ significantly within participants between sessions (Fig. 5A,B_(i; iii)_). While within participant power modulation did not differ significantly between sessions at the sensor level (Fig. 5A_(ii)_), corresponding source spectral modulation was significantly larger in the 2nd compared to the 1st session (t_12_ = − 3.391, p < 0.001), and this difference appeared to be driven by a subset of participants (Fig. 5B_(ii)_). We observed significant within participant sensor vs. source differences (t_12_ = − 3.370; t_12_ = − 3.306, for sessions 1 and 2 respectively; p < 0.01) for peak frequency of induced oscillations despite small average differences. In line with observed spectral responses (Fig. 3), power modulations corresponding to peak frequencies were significantly lower (p < 0.01) at sensor compared to source level (t_12_ = − 3.391; t_12_ = − 3.858, for sessions 1 and 2 respectively; Table 2). Finally, there were no significant differences in the corresponding peak frequency bandwidth derived from sensor and source for each session.

**Fig. 6:**
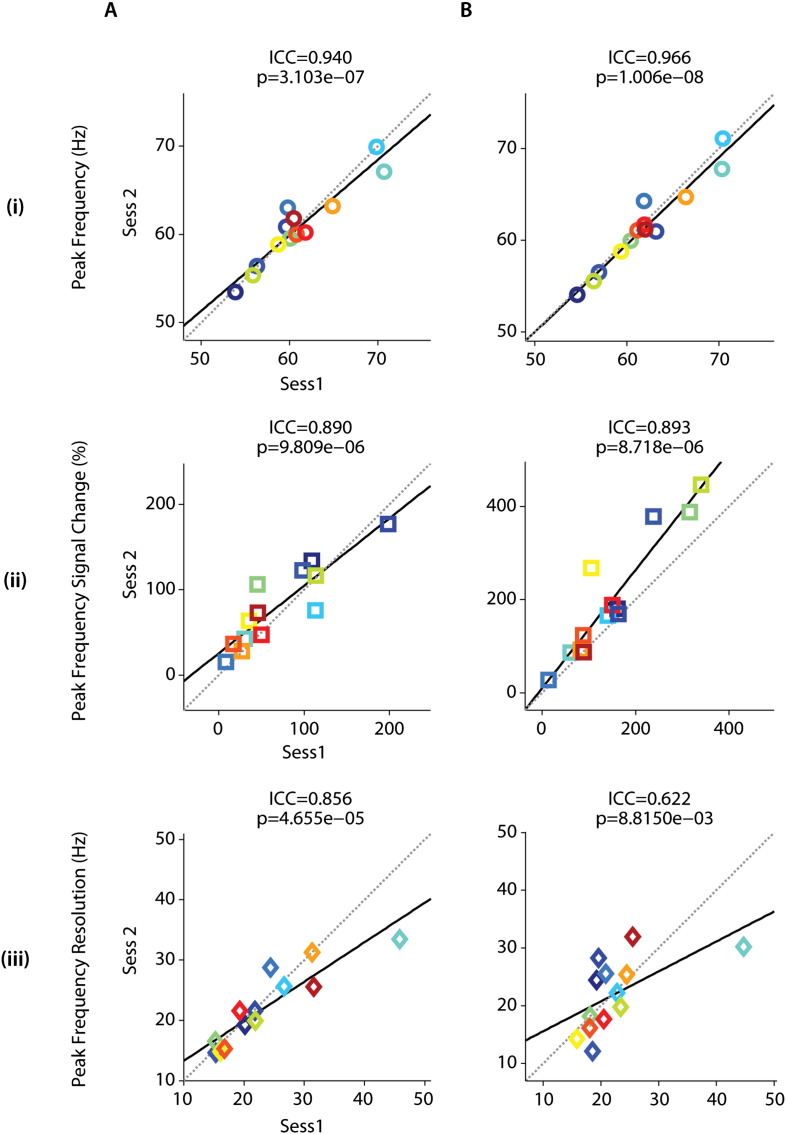
Summary of intraclass correlations (ICCs). Test–retest reliability was assessed with ICCs and the corresponding results are summarized for sensor (A) and source (B) derived (i) peak frequency as well as corresponding (ii) signal change and (iii) frequency bandwidth.

ICC values for peak frequency, corresponding spectral signal change and peak frequency bandwidth measures indicated overall strong reliability (mean ICC = 0.861; ICC range: 0.622–0.966; p < 0.001; refer to Table 3 and Fig. 6) and comparable sensor- (mean ICC = 0.895) and source-derived (mean ICC = 0.827) oscillatory measures. ICC values were highest for peak frequency, followed by corresponding spectral signal change and frequency bandwidth, for both sensor and source derived measures. Assessment of peak frequency yielded strong reliability (ICC > 0.90) for both sensor (ICC_PEAK-FREQ_ = 0.940; p < 0.0001) and source (ICC_PEAK-FREQ_ = 0.966; p < 0.0001) derived peak frequencies (Fig. 6A_(i)_, B_(i)_). Similarly, we observed high ICC values for both sensor (ICC_SPECTRAL_ = 0.890; p < 0.0001) and source (ICC_SPECTRAL_ = 0.893; p < 0.0001) derived spectral modulations at peak frequencies (Fig. 6A_(ii)_, B_(ii)_). The bandwidth of peak frequency (Fig. 6A_(iii)_, B_(iii)_) yielded higher reliability for sensor (ICC_FREQ-RES_ = 0.856; p < 0.0001) compared to source derived (ICC_FREQ-RES_ = 0.622; p < 0.01) signals.

Beyond the mean cluster signals derived from our maximally modulated visual voxels, we observed (Supplementary Figs. SFig. 5_(i)_ and SFig. 5_(ii)_) narrow ranges of peak frequencies (SFig. 5_(i)__(ii)_, A) across visual cortical regions in the range comparable to those found for the maximally task-induced voxel clusters (Fig. 4A, B_(i)_). We also noted a consistent trend that the majority of visual voxels have lower spectral signal changes, with a smaller set of voxels yielding the largest signal changes (SFig. 5_(i)__–(ii)_, B). Supplementary Figs. SFig. 6_(i)_ and SFig. 6_(ii)_ provide a 3D overview of the variability of peak frequencies derived for each participant's ~ 800 AAL-labeled visual cortical voxels in both sessions (SFig. 6_(i)__–(ii)_, A). We noted the variability in signals of correspondent AAL-parceled voxels between sessions and the overlap of maximally task induced voxel clusters with visual cortical voxels that generally manifested high signal changes (SFig. 6_(i)_, SFig. 6_(ii)_, B, C). Furthermore, assessment of peak frequencies and spectral signal changes indicated similar distributions within each AAL-parceled visual cortical region (SFig. 7_(i)–(xiii)_). Additionally, voxels manifesting the largest spectral signal changes tended to be found within the calcarine, lingual gyri, and on occasion, the occipital lobules.

## Discussion

The present study assessed the test–retest reliability of visually induced high-frequency (30–100 Hz) oscillations derived from MEG sensor and estimated source signals. Overall, estimates of individual peak gamma frequencies, spectral modulation and peak frequency bandwidth were remarkably stable across measurements. Although the spectral signal change was stronger in the repeated session for source-derived peak frequency spectra, both sensor and source spectral modulations exhibited comparably high repeatability. Peak oscillatory frequency yielded highest measure of reliability followed by its corresponding spectral signal change and peak frequency bandwidth. These stability measures of induced gamma oscillatory activity corroborate those repeatability assessments at source level reported by Muthukumaraswamy et al. (2010) for static visual stimulus. Importantly, the current study suggests that spectral estimates of both sensor and source derived parameters are both highly reliable. In addition, the current study suggests that the source-space approaches significantly improve SNRs of high frequency oscillations.

Consistent with previous findings (Hoogenboom et al., 2006, Muthukumaraswamy et al., 2010, Schwarzkopf et al., 2012), measures of visually-induced high frequency oscillations manifested individual variability, particularly for peak frequency and its corresponding spectral modulation. Previous study by Schwarzkopf et al. (2012) revealed higher peak gamma frequency being associated with larger primary visual cortical surface area, and suggested that differences in visually induced peak gamma frequency could be attributed to individual differences in the structural and functional architecture of visual cortex. Interestingly, cortical environments that are highly similar in e.g. receptor density, cytoarchitecture and/or coupling strength are thought to enable greater consistency in oscillatory activity (e.g. Breakspear et al., 2010). These observations led Schwarzkopf et al. (2012) to further suggest the role of lateral intra-areal inhibitory processes (Alitto and Dan, 2010, Edden et al., 2009) in sharpening sensory responses. Relevantly, the narrow range of individual peak gamma frequency observed across the extent of visual cortical areas assessed may arise from synchrony-enhancing mechanisms of interneuronal dendritic gap junctions in spatially extended interneuron networks (Traub et al., 2001). On the other hand, theoretical studies (Cannon et al., 2014, Serenevy and Kopell, 2013) have indicated that heterogeneous cell properties and their connections (e.g. those that allow inputs to arrive at target networks with a range of phases covering a large part of the gamma cycle) may facilitate network entrainment (e.g. through differential recruitment of fast-spiking interneurons) that might otherwise not be feasible if driving phases were highly similar. It is therefore reasonable to infer that neural oscillations at any given frequency (and brain region) may arise from various mechanisms (e.g. Ainsworth et al., 2012).

As with previous study by Muthukumaraswamy et al. (2010) we observed that maximally modulated voxels do not necessarily manifest within the exact location in the repeated session. However, the 3-dimensional Euclidean distance between maximal loci suggested close proximities given an imaging resolution of 6 mm. Furthermore, spectral modulation within the visual areas reflected some inhomogeneity, as averaging across all (a priori) visual voxels led to the loss of signal. This inference corroborated with findings from our assessment of peak frequencies of individuals' voxels within the visual cortical regions, revealing that a majority of voxels manifested lower spectral modulations relative to a small proportion of voxels exhibiting the strongest (within participants) spectral signal change. Additionally, corresponding AAL-parceled voxels revealed between session variability in spectral signal change. These observations highlight the challenges in extracting signals from source as well as the likely mixing of signals of differing strengths that are picked up by the MEG sensors. In particular, the strength of MEG source signals is dependent on its orientation, increasing as the source orientation deviates from the radial towards the tangential axis (Hämäläinen et al., 1993). These are likely to contribute to the variability in spectral signal change and bandwidth corresponding to the induced peak gamma frequency both across participants and between sessions. Additionally, varying beamforming algorithms and approaches employed in previous (e.g. Muthukumaraswamy et al., 2010, Schwarzkopf et al., 2012) and current research may have also contributed to the differences in the reported spectral modulation. Employing a broad-band time-frequency decomposition with multi-tapers to extract the maximally modulated voxels and subsequent finer-resolution source signals spectral decomposition, we were able to recover the spectral modulations observed in sensor-derived signals or exceeded them at source level.

Interestingly, we observed that gamma spectral modulation was relatively higher in the repeated session for many participants, particularly at the source level. It has previously been shown that gamma band activity is enhanced in neurons driven by attended stimuli (Fries et al., 2001) and associated with corresponding improvements in perceptual performance (e.g. Taylor et al., 2005, Womelsdorf et al., 2006). With repeated performance of the same visual task, our participants became faster (although not significantly so) and more accurate in detecting stimulus acceleration. More recently, neurophysiological studies by Ray et al. (2013) and Chalk et al. (2010) highlighted that where attention is called upon in a task, the modulation in gamma spectral power may be a reflection of the changes in the underlying excitation-inhibition activity, which could be accounted for by normalization. The normalization model of attention posits that a neuron's (gamma) response is suppressed by the overall response of its neighboring neurons and predicts that attention increases its excitatory drive, which in turn increases normalization. Crucially, the findings of Ray et al. (2013) demonstrated that even with attentional load fixed, increased normalization, e.g. when a cell's receptive field processes its non-preferred (vs. preferred) motion direction, led to an increase in gamma spectral power. It is conceivable that as the visual task becomes more familiar it calls upon less directed attention towards the center fixation spot for adequate performance. From this perspective, and the variable maximally modulated voxel location, subtle modulation in participants' focal attention and corresponding normalization could underlie the differing excitability that yielded varying induced gamma signal change between MEG sessions. Although beyond the scope and resolution of the present analysis, changes in signal strength between testing sessions might be induced through stimulus repetition. Recent study in primate visual cortex (Brunet et al., 2014) reported that repeated presentation of a pair of orthogonal visual gratings during a change detection task was associated with increased local and inter-areal gamma activity, and suggested the role of lateral inhibitory activity in sharpening the underlying stimulus representation. Interestingly, the authors further noted a corresponding increase in peak gamma frequency with stimulus repetition and observed enhanced synchrony, which is not observed in the present study.

Last but not least, it has been reasoned that source analysis may yield more reliable estimates of MEG-activity compared to sensor-derived measures because exact positioning of participants under the MEG sensors across repeated recordings is not a prerequisite. Furthermore, the beamforming approach in source analysis acts as spatial filters in suppressing background activity that may lower the reliability of sensor signals and thereby improve the signal estimates. Here, we replicated the findings observed in our previous reliability assessment of auditory steady state responses from sensor and source derived signals (Tan et al., 2015), which highlighted that even without available continuous head position information (e.g. Deuker et al., 2009) careful monitoring of head and sensors' positioning can yield highly reliable estimates of oscillatory measures from MEG sensor signals. We note that source estimated spectral modulation in some participants exceeded those derived from sensor signals. However, relative to sensor-derived estimates source-derived measures yielded less consistency in its corresponding spectral bandwidth. Importantly, our findings affirm that peak frequency measures of gamma-band brain oscillations can be very reliably estimated from sensor (van Pelt et al., 2012) and source (Muthukumaraswamy et al., 2010) derived MEG signals. Additionally, our assessments indicated that while spectral signal changes are more variable across visual cortical areas, individual peak gamma frequency manifested a narrow range across large areas of the visual cortex.

## Conclusion

The present study further substantiates the view that MEG-derived oscillatory signatures of visual (and other sensory) responses are highly reproducible. This finding is important as MEG is increasingly used as a tool for the identification of biomarkers in clinical research (e.g. Georgopoulos et al., 2010, Sun et al., 2013) and for investigation of rhythmic activity during normal brain functioning. Specifically, our research underscores peak frequency of visually induced brain oscillations as particularly reliable. Given that gamma oscillation is generated by well-coordinated inhibitory and/or excitatory neuronal networks (Buzsáki and Wang, 2012) and peak frequency of induced visual gamma has been shown to decrease with age (Gaetz et al., 2012, Muthukumaraswamy et al., 2010), estimates of peak gamma frequency could be useful in tracking individual's underlying neural excitability (Ray and Maunsell, 2015) over time. No doubt, further studies are needed to better link macroscopic measures of gamma activity e.g. M/EEG with quantifiable proxies of microscopic (e.g. molecular; network-level) processes. Nonetheless, these insights are particularly encouraging for larger-scale, longitudinal, and/or clinical studies that require repeated MEG measurements, and for which study outcomes are not contingent on source-derived oscillatory readouts.

## Figures and Tables

**Table 1:** Location, anatomical label(s) and corresponding T-statistics of participants' maximally modulated visual voxels, determined by non-parametric source-statistics in each MEG session. See Methods and Results for further details.

	Session 1					Euclidean dist. (mm)	Session 2				
	Anatomical label (AAL)	SPM coordinates (mm)			T-stats		T-stats	SPM coordinates (mm)			Anatomical label (AAL)
		x	y	z				x	y	z	
S1	Right lingual gyrus | right inferior occipital lobule	24	− 90	− 14	4.953	54	5.090	− 30	− 90	− 8	Left inferior occipital lobule | left middle occipital lobule
S2	Right calcarine | right lingual gyrus	12	− 102	− 8	7.258	15	8.578	18	− 90	− 14	Right lingual gyrus
S4	Right lingual | right cerebellum crus1	18	− 96	− 26	5.099	6	5.654	18	− 96	− 20	Right lingual gyrus
S5	Right cuneus | right superior occipital lobule | right superior parietal lobule	24	− 84	46	3.330	12	5.166	12	− 84	46	Right cuneus | right superior parietal lobule | right precuneus
S6	Left lingual gyrus | left inferior occipital lobule	− 18	− 96	− 14	6.647	15	7.742	− 12	− 102	− 2	Left calcarine | left middle occipital lobule
S7	Right calcarine | right cuneus	12	− 96	4	5.659	27	6.326	− 12	− 108	4	Left middle occipital lobule
S8	Left calcarine | left middle occipital lobule	− 6	− 108	− 2	7.959	6	9.134	− 6	− 108	4	Left middle occipital lobule
S9	(Right) calcarine	6	− 102	− 2	6.257	6	6.885	6	− 102	− 8	Left calcarine
S10	Left middle occipital lobule	− 18	− 96	4	4.882	25	6.275	6	− 90	4	(Right) calcarine
S11	Left | right calcarine	0	− 102	− 2	6.993	12	6.282	12	− 102	− 2	Right calcarine
S12	Left lingual gyrus	− 12	− 90	− 14	5.900	48	5.626	24	− 102	16	Right superior occipital lobule
S13	Left calcarine | left cuneus	− 6	− 84	16	8.095	21	8.910	− 18	− 96	28	Left superior occipital lobule
S14	Left calcarine | left cuneus | left middle occipital lobule	− 6	− 102	4	7.006	26	8.930	18	− 96	− 2	Right calcarine

**Table 2:** Summary of participants' oscillatory measures – peak frequency (Hz), spectral signal change (%), peak frequency bandwidth (Hz) – for each MEG session at sensor and source levels. Within participant statistical comparisons of sensor vs. source measures highlight significant differences (p < 0.05). Refer to Results for further details.

Session		Sensor-level		Source-level				Sensor vs. source	
		1	2	1	2			1	2
Peak frequency (Hz)	Mean	61.0	61.7	61.9	61.3		t_12_	− 3.370	− 3.306
	± SD	4.9	4.5	4.9	4.8		*p*	0.006	0.006
								
Spectral signal change (%)	Mean	68.7	79.8	149.7	199.9	⁎	t_12_	− 3.391	− 3.858
	± SD	54.3	48.2	96.9	131.5		*p*	0.005	0.002
								
Frequency bandwidth — FWHM (Hz)	Mean	23.6	22.2	23.5	22.0		t_12_	1.250	0.139
	± SD	8.7	6.3	7.2	6.2		*p*	0.235	0.891

⁎ Paired t-test significant difference (p < 0.001).

**Table 3:** Summary of reliability assessments. Oscillatory variables: peak frequency (Hz), and spectral signal change (%) show comparably high intraclass correlations (ICCs) at sensor and source levels. Peak frequency bandwidth (Hz) yielded higher reliability with sensor vs. source derived signals.

		Sensor				Source		
		ICC	LB	UB		ICC	LB	UB
Peak frequency (Hz)	*r*	0.940	0.815	0.981	*r*	0.966	0.893	0.990
	*p*	3.10E-07			*p*	1.01E-08		
							
Spectral signal change (%)	*r*	0.890	0.681	0.965	*r*	0.893	0.687	0.966
	*p*	9.81E-06			*p*	8.72E-06		
							
Frequency bandwidth — FWHM (Hz)	*r*	0.856	0.595	0.954	*r*	0.622	0.133	0.867
	*p*	4.65E-05			*p*	8.82E-03		

## Appendix A Supplementary data

SFig. 1 Experimental paradigm. Each trial began with an initial presentation of a central fixation spot (500 ms) whose contrast is subsequently reduced by 40% for a further 1500 ms, prompting the upcoming moving grating presentation. The ensuing concentric moving grating contracted towards the fixation point and accelerated randomly between 750–3000 ms post grating onset. Participants indicated the detection of this acceleration with a button press within 500 ms of its occurrence. Response feedback was provided during the inter-trial interval (1000 ms). Rare incidences (10%) in which no acceleration occurred were interspersed within a series of 80 trials that made up a block of the visual task. Refer to Methods for further details. SFig. 2 Maximally modulated voxels derived for all participants in both experimental sessions. Each participant's maximally modulated voxels, as determined by non-parametric T-statistics, including their surrounding 26 voxels (Fig. 2_(iv)_) are interpolated onto their corresponding MNI-normalized brain volumes. For each session, each participant's maximally modulated voxels in response to moving stimulus grating are shown (from left to right) in coronal, sagittal and axial views. The majority of participants' maximal modulated voxel are located and clustered within the visual cortex. See Methods and Results for further details. SFig. 3 Grand average visually induced responses for each MEG session. Power signal change relative to baseline is averaged across participants separately for signals derived at sensor (i, ii) and at source (iii, iv) for both MEG sessions. Topographies of relative power change for MEG sessions 1 (A) and 2 (B) are shown for (i) all sensors and for (ii) only visual sensors. These signal change topographies are averaged across all participants (i, ii) and aligned on LHS over frequency axes. (ii) Topographies for both the duration of interest 0.5–2 s and frequency range of 50–70 Hz are shown on the RHS above the time-frequency plots. Grand average spectral modulation within visual voxels (iii) and in voxels yielding maximal sensory modulations (iv) is shown. The spectral topography plots (i, ii) highlight that the main contribution for the observed spectral response from all sensors (i) is clustered within the visual-sensors (ii) chosen a priori for the sensor-level analyses. Furthermore, spectral modulation was enhanced in sources and maximally modulated relative to baseline (iv), compared to visual-region-specific source spectral modulations (iii). SFig. 4 Analysis-approach for deriving visually induced response parameters of interest. Overview of analysis-approach illustrated with data from 1 participant (S01). For each participant, power signal change relative to baseline was derived at both sensor (i) and source (ii), and for each MEG session (session 1 — upper, and session 2 — bottom rows, respectively). (A) Time-frequency plots for signal change, (B) the corresponding baseline (green) and stimulus-related (red) signals are depicted for both MEG sessions (C). Subsequently, (D) Gaussian fitting of time series was performed for both MEG sessions (solid = average frequency signal change time series; dotted = fitted Gauss for sessions 1 and 2, respectively), from which the parameters: peak frequency, the corresponding signal change and frequency bandwidth were extracted for reliability assessment. Time range for plot illustration is from − 1 to 1.75 s. Refer to Methods and Results for further details. SFig. 5 Distributions of peak frequency and spectral modulation of visual cortical voxels. Histograms of peak frequency (A) and spectral signal change (B) derived from each individual's (~ 800 voxels within) AAL-parcelled visual cortical areas are shown for both MEG sessions and for all participants: S01, S02, S04–S08 (i) and S09–S14 (ii). SFig. 6 Individual visual cortical voxel's peak frequency as a function of spectral modulation. The peak frequency derived for each individual participant's (~ 800) AAL-parcelled visual cortical voxel is shown as a function of corresponding spectral signal change with reference to AAL-parcelled voxel labels (A). The peak frequency and signal change derived from any maximally task-induced cluster of voxels corresponding to the AAL-parcelled visual cortical voxels are overlaid onto each participant's plots (S01, S02, S04–S08 (i) and S09–S14 (ii)) of peak frequency and spectral signal change for MEG sessions 1 (B) and 2 (C). The bins and tick-marks of peak frequency on the x-axis in (B, C) follow the histogram binning in SFig. 5A, which differs from SFig. 6A. SFig. 7 Distributions of peak frequency and spectral modulation by AAL-parcelled visual regions. Histograms of peak frequency (A) and spectral signal change (B) derived from each individual's (~ 800 voxels within) AAL-parcelled visual cortical areas are shown for both MEG sessions and for all participants: (i)–(xiii). Image 1
