# Supplementary material for: MEG sensor and source measures of visually induced gamma-band oscillations are highly reliable
Source: Neuroimage. 2016 Aug 15;137:34–44. doi: 10.1016/j.neuroimage.2016.05.006 (PMC5405052; doi:10.1016/j.neuroimage.2016.05.006)
Supplement: Supplementary file 1 — SFig. 1 Experimental paradigm. Each trial began with an initial presentation of a central fixation spot (500 ms) whose contrast is subsequently reduced by 40% for a further 1500 ms, prompting the upcoming moving grating presentation. The ensuing concentric moving grating contracted towards the fixation point and accelerated randomly between 750–3000 ms post grating onset. Participants indicated the detection of this acceleration with a button press within 500 ms of its occurrence. Response feedback was provided during the inter-trial interval (1000 ms). Rare incidences (10%) in which no acceleration occurred were interspersed within a series of 80 trials that made up a block of the visual task. Refer to Methods for further details. SFig. 2 Maximally modulated voxels derived for all participants in both experimental sessions. Each participant's maximally modulated voxels, as determined by non-parametric T-statistics, including their surrounding 26 voxels (Fig. 2(iv)) are interpolated onto their corresponding MNI-normalized brain volumes. For each session, each participant's maximally modulated voxels in response to moving stimulus grating are shown (from left to right) in coronal, sagittal and axial views. The majority of participants' maximal modulated voxel are located and clustered within the visual cortex. See Methods and Results for further details. SFig. 3 Grand average visually induced responses for each MEG session. Power signal change relative to baseline is averaged across participants separately for signals derived at sensor (i, ii) and at source (iii, iv) for both MEG sessions. Topographies of relative power change for MEG sessions 1 (A) and 2 (B) are shown for (i) all sensors and for (ii) only visual sensors. These signal change topographies are averaged across all participants (i, ii) and aligned on LHS over frequency axes. (ii) Topographies for both the duration of interest 0.5–2 s and frequency range of 50–70 Hz are shown on the RHS above the time-fre [file mmc1.pdf]

MEG sensor and source measures of visually induced gamma-band oscillations are highly reliable

H.-R.M. Tan* , J. Gross , P.J. Uhlhaas

Centre for Cognitive Neuroimaging (CCNi), Institute of Neuroscience and Psychology (INP),
College of Medical, Veterinary and Life Sciences, College of Science and Engineering,
University of Glasgow, 58 Hillhead Street, Glasgow G12 8QB, United Kingdom

*** Corresponding author**

Heng-RuMay.Tan@glasgow.ac.uk | hengrumay@gmail.com

Please cite this article + supplementary data as: **Tan, H.-R.M. et al., MEG sensor and source measures of visually induced gamma-band oscillations are highly reliable, *NeuroImage* (2016), http://dx.doi.org/10.1016/j.neuroimage.2016.05.006**

Appendix A. Supplementary data

Supplementary figures SFig.1—7 and corresponding figure captions are provided in this document.

SUPPLEMENTARY FIGURES:

SFig. 1

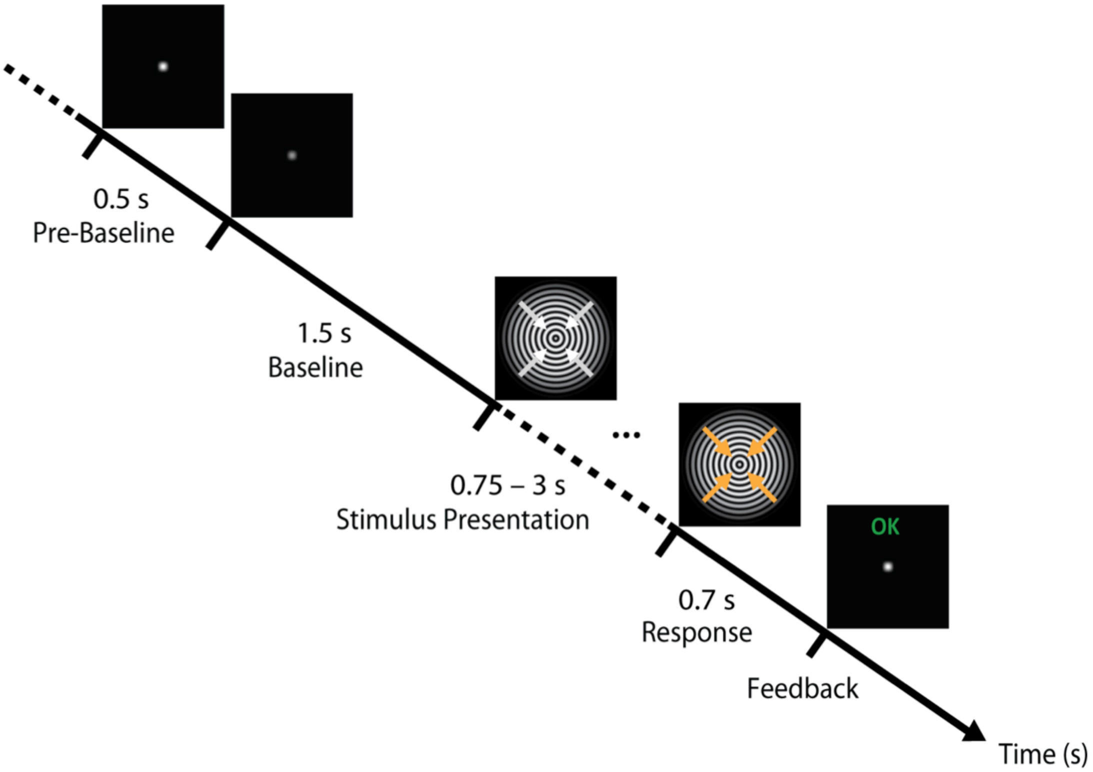

SFig. 2

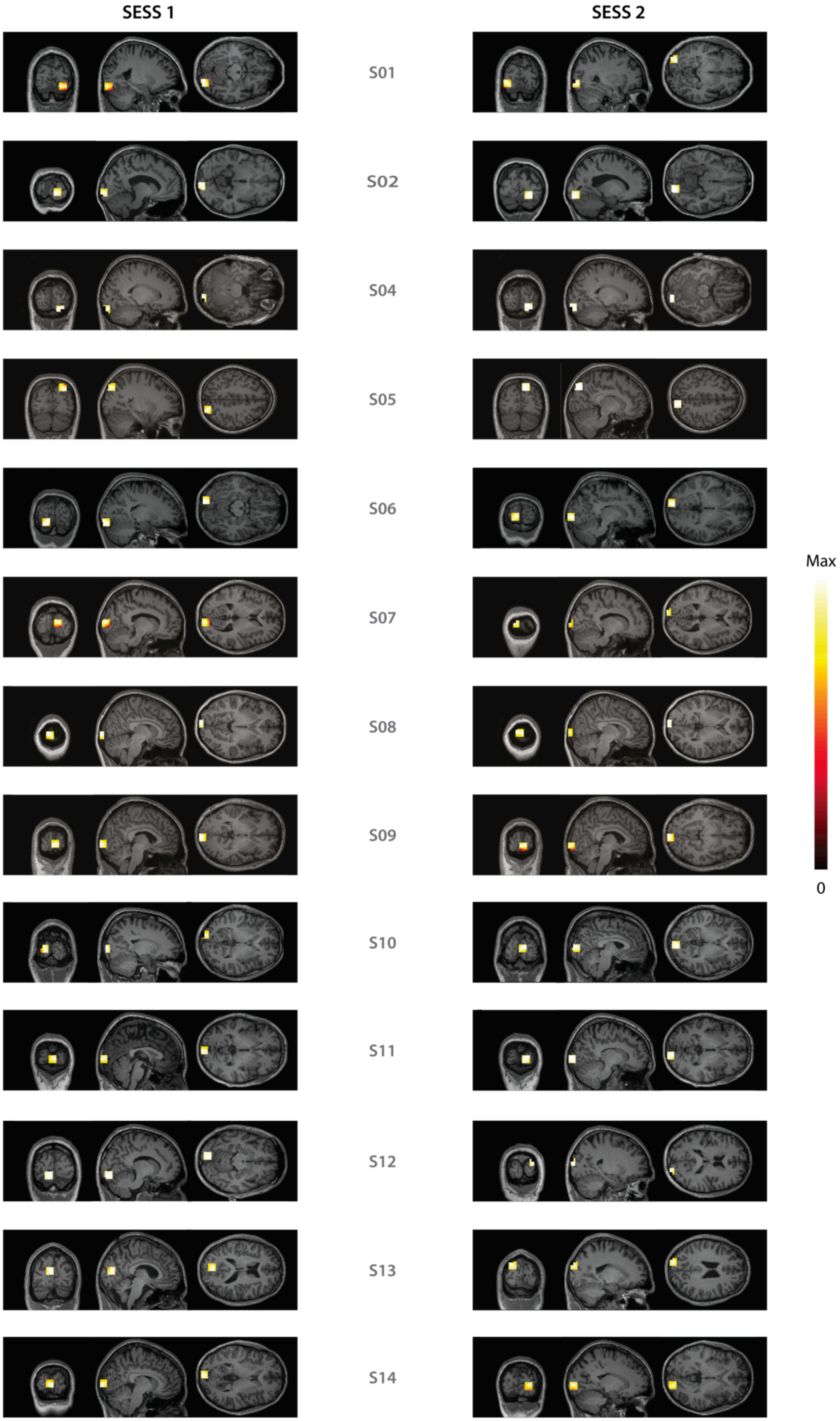
SESS 1
SESS 2
S01
S02
S04
S05
S06
S07
S08
S09
S10
S11
S12
S13
S14
Max
0

SFig. 3

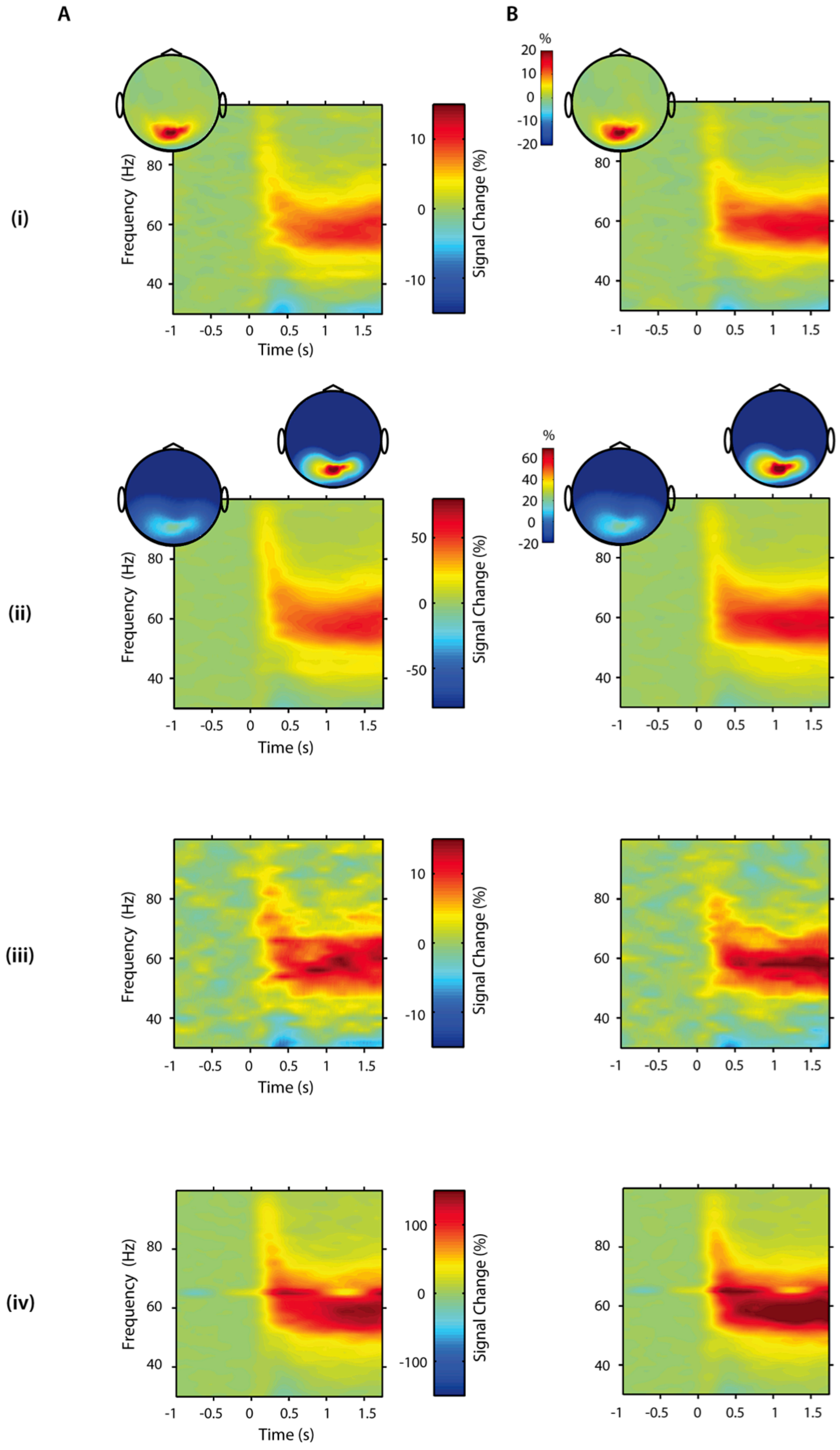
A
B
(i)
(ii)
(iii)
(iv)
Frequency (Hz)
Time (s)
Signal Change (%)
%
-1 -0.5 0 0.5 1 1.5
40 60 80

SFig. 4

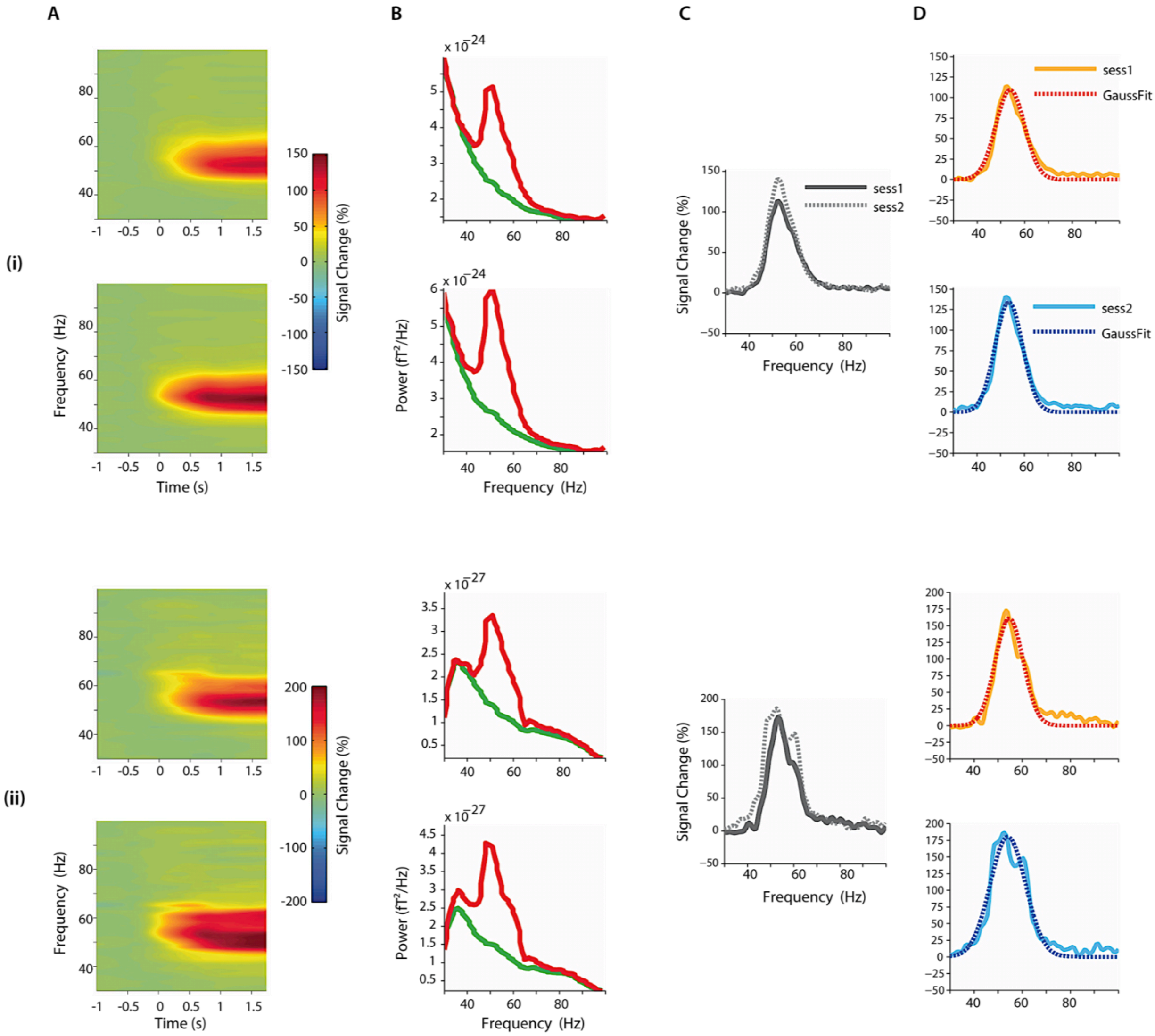
A
B
C
D
(i)
(ii)
Frequency (Hz)
Time (s)
Signal Change (%)
Power (fT²/Hz)
Frequency (Hz)
x 10⁻²⁴
x 10⁻²⁷
sess1
sess2
GaussFit

SFig. 5

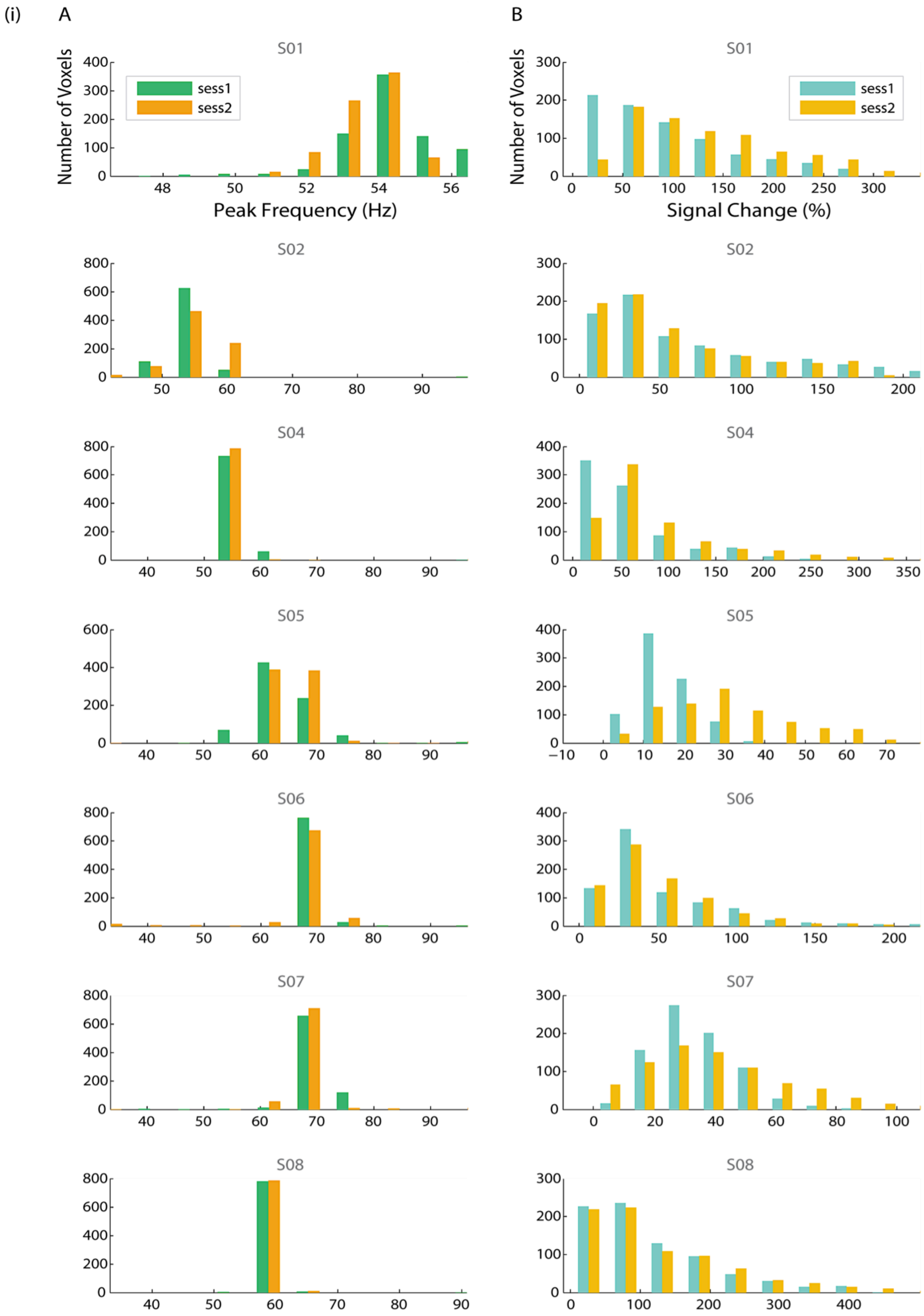
(i)
A
B
S01
Number of Voxels
sess1
sess2
Peak Frequency (Hz)
Signal Change (%)
S02
S04
S05
S06
S07
S08

SFig. 5

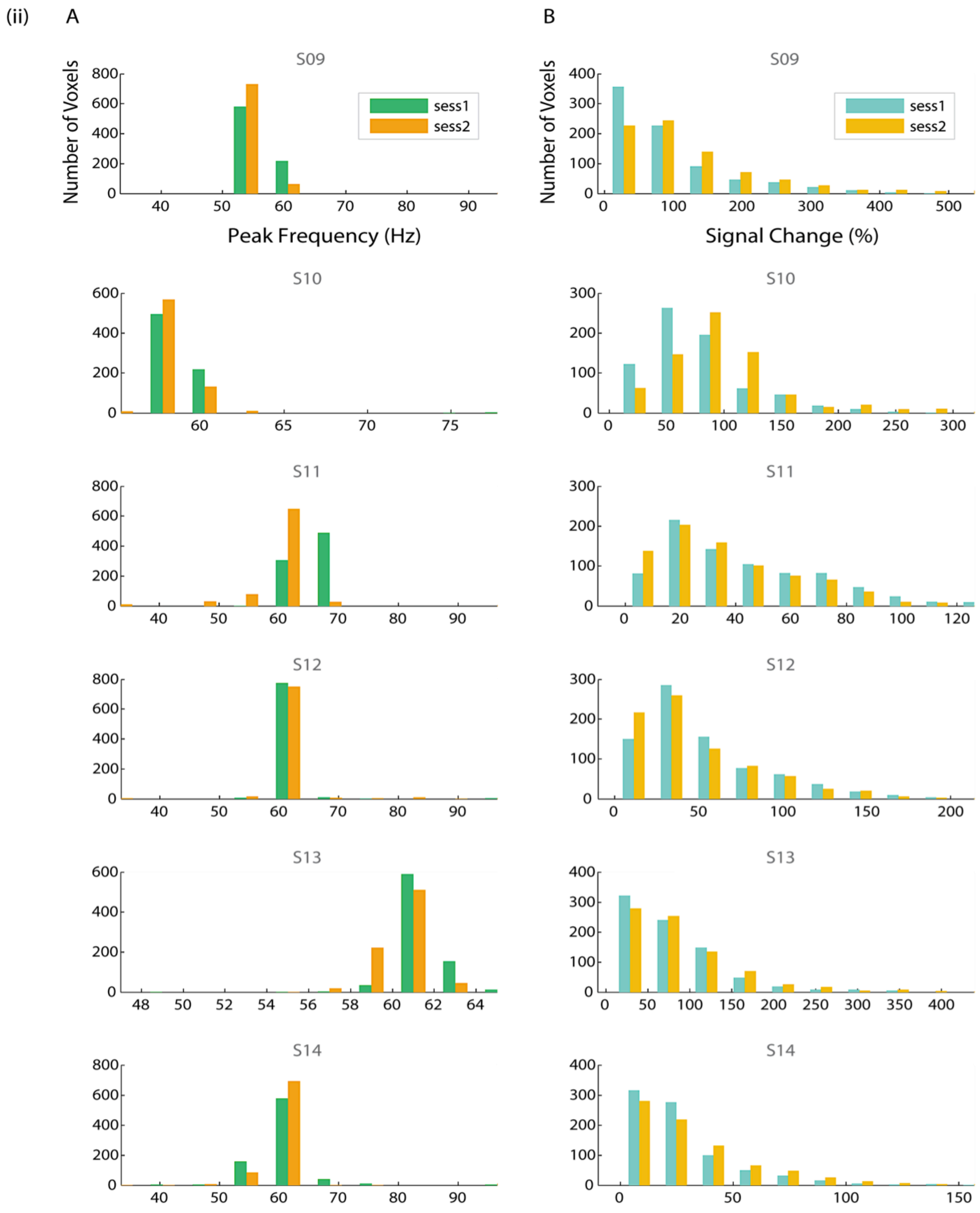

http://dx.doi.org/10.1016/j.neuroimage.2016.05.006

SFig. 6

(i)

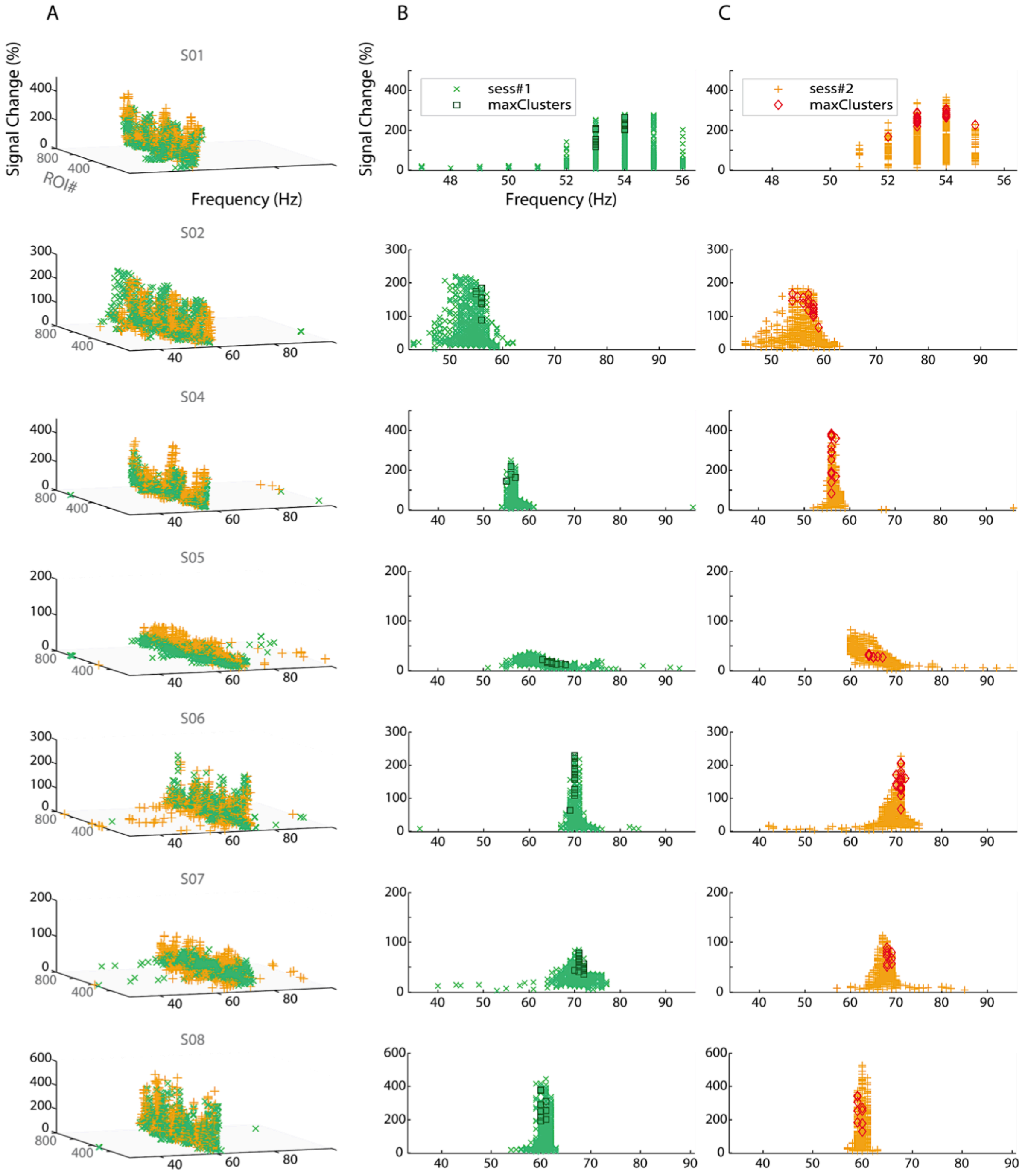
A
B
C
S01
S02
S04
S05
S06
S07
S08
Signal Change (%)
ROI#
Frequency (Hz)
sess#1
maxClusters
sess#2
maxClusters

SFig. 6

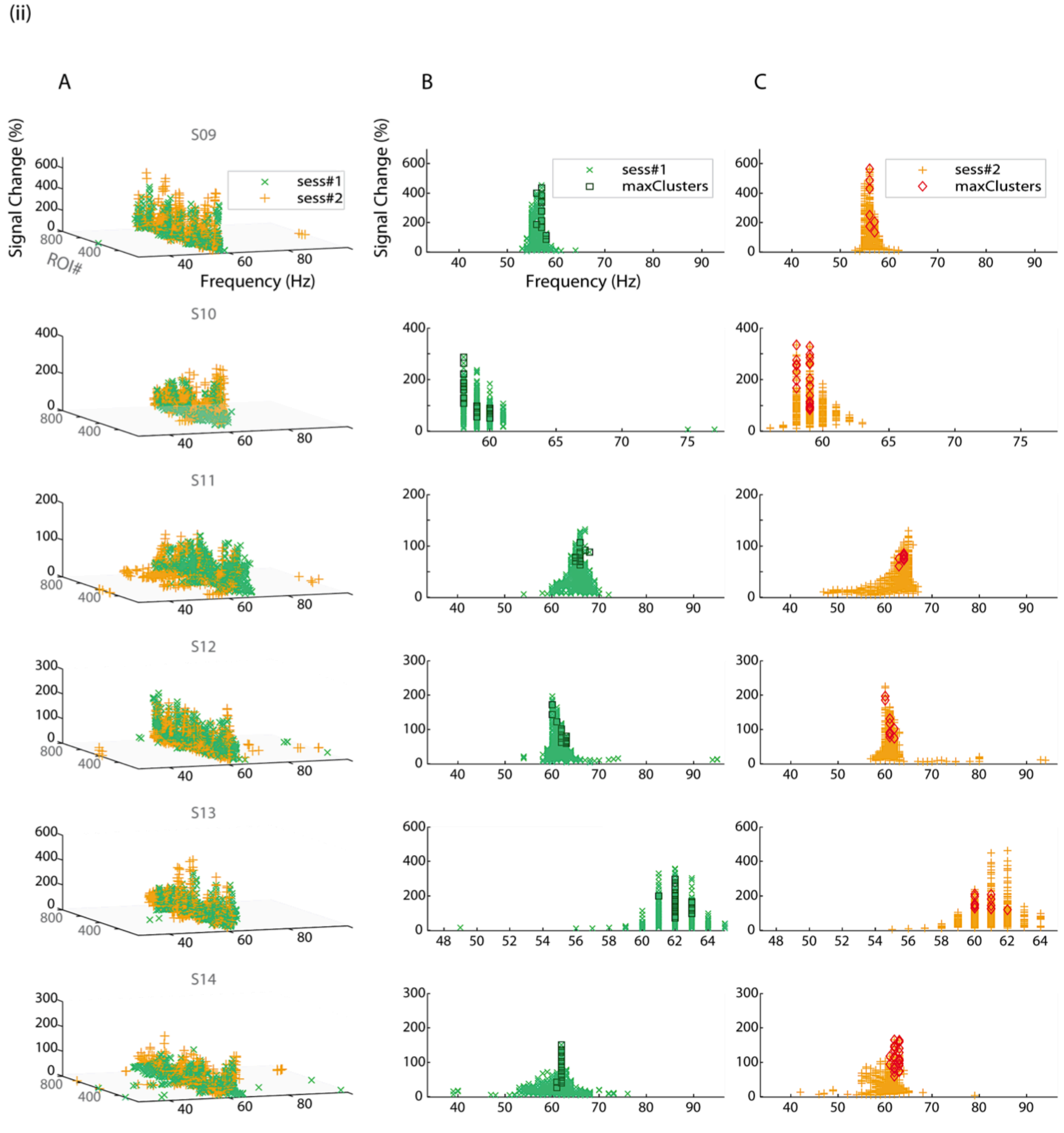
(ii)
A
B
C
S09
S10
S11
S12
S13
S14
Signal Change (%)
Frequency (Hz)
ROI#
sess#1
sess#2
maxClusters

SFig. 7

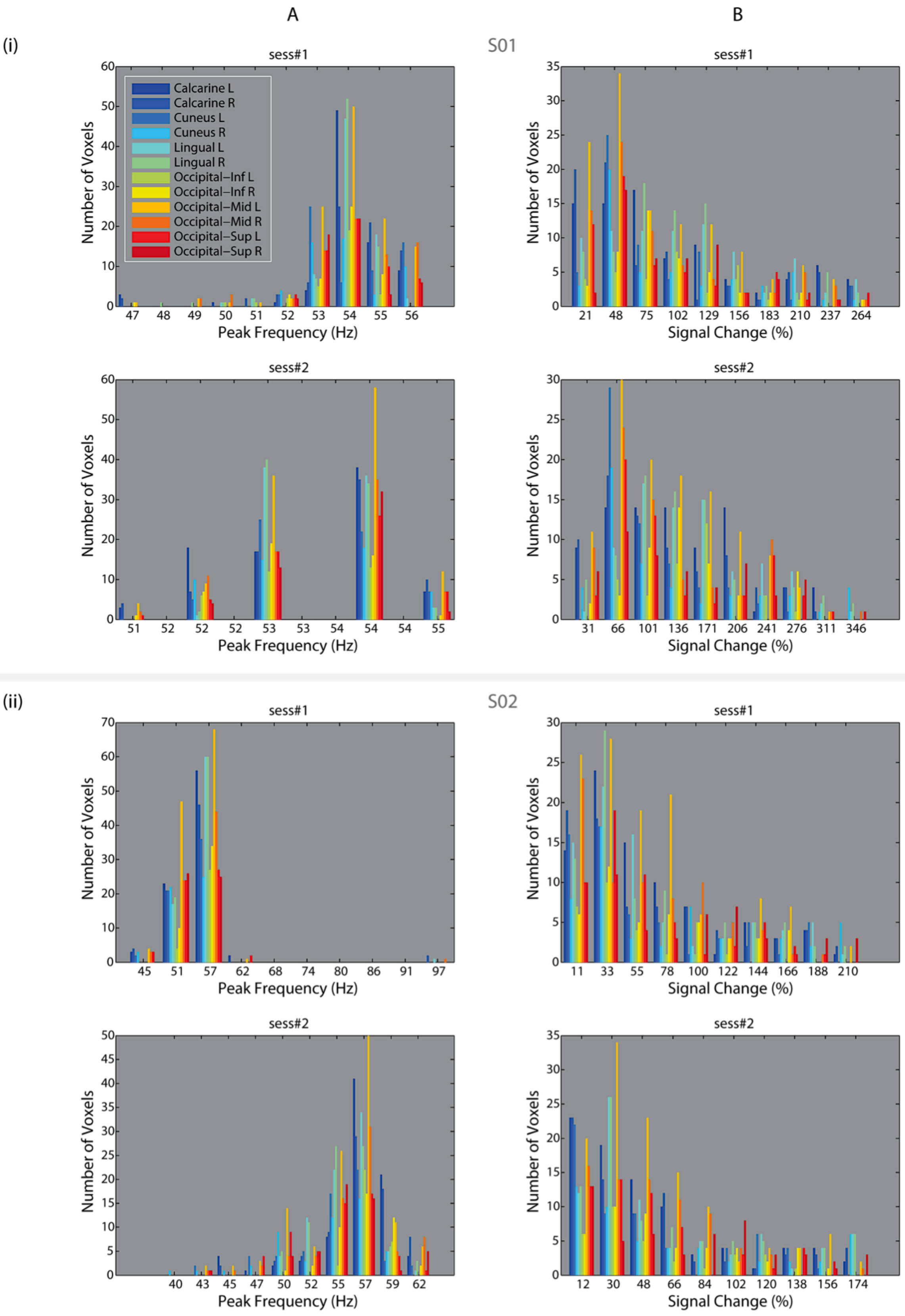

http://dx.doi.org/10.1016/j.neuroimage.2016.05.006

SFig. 7

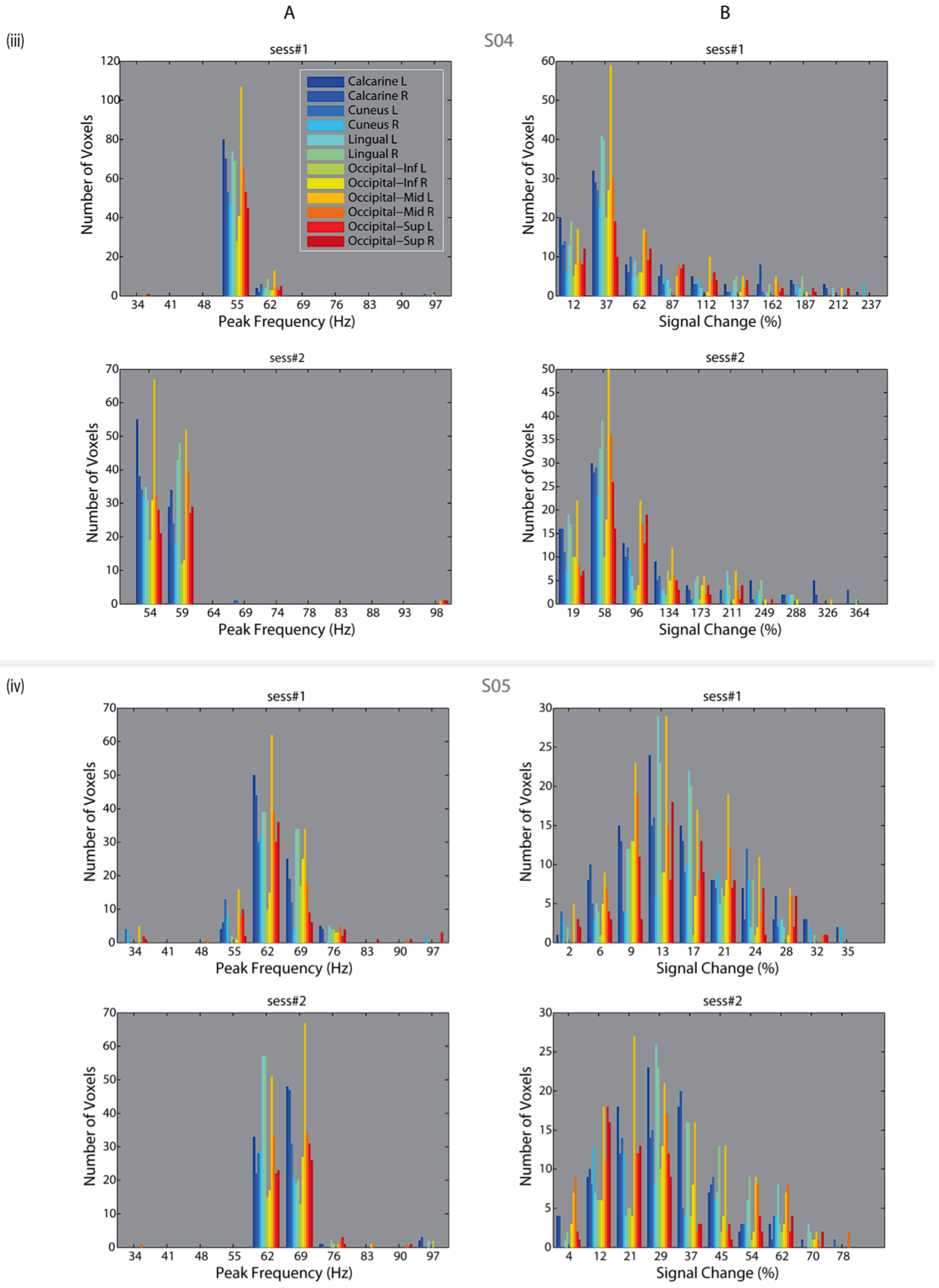
A
B
(iii)
S04
sess#1
Calcarine L
Calcarine R
Cuneus L
Cuneus R
Lingual L
Lingual R
Occipital–Inf L
Occipital–Inf R
Occipital–Mid L
Occipital–Mid R
Occipital–Sup L
Occipital–Sup R
Number of Voxels
Peak Frequency (Hz)
Signal Change (%)
sess#2
(iv)
S05
sess#1
sess#2
Number of Voxels
Peak Frequency (Hz)
Signal Change (%)

SFig. 7

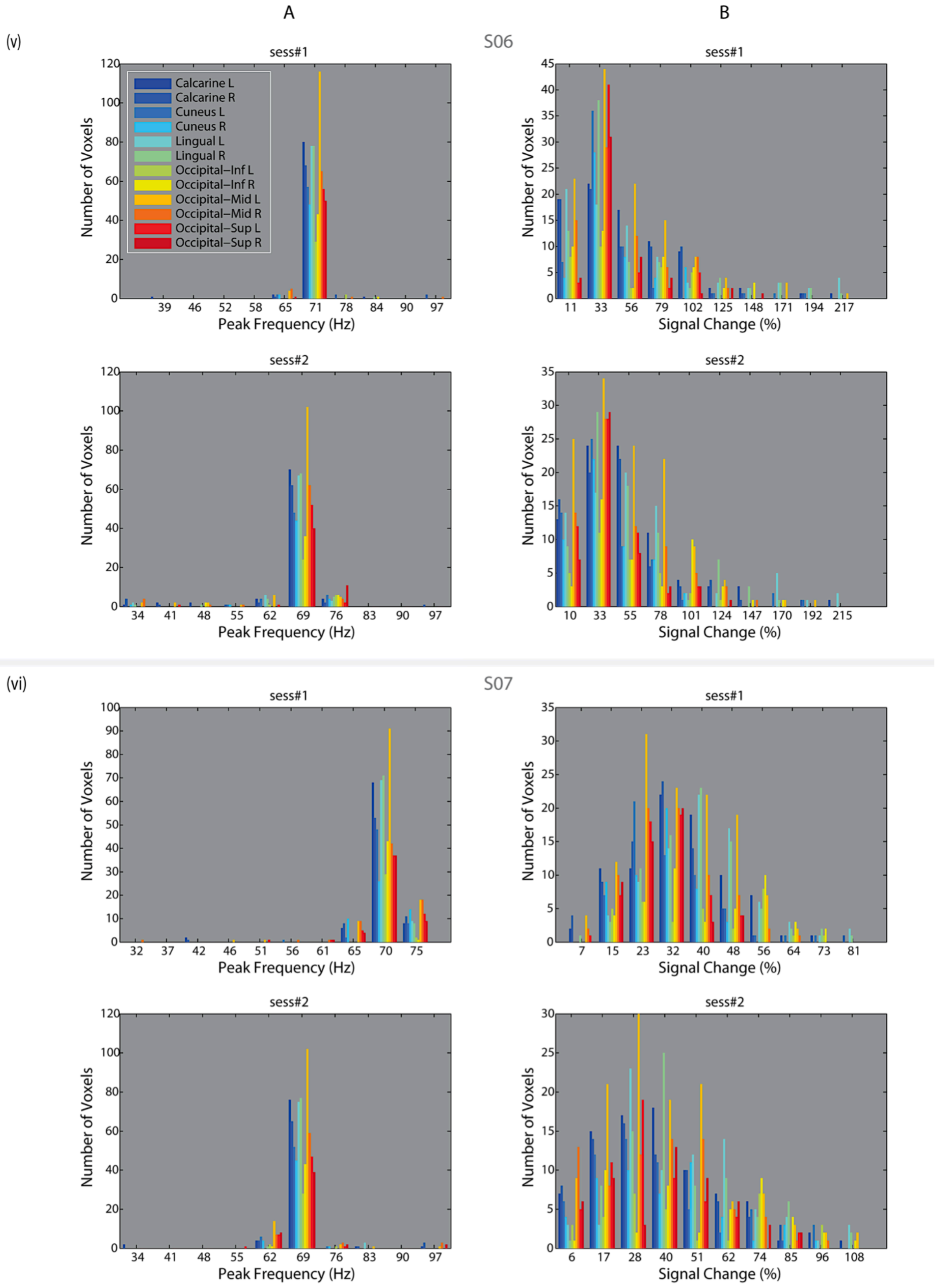
A
B
(v)
S06
sess#1
Calcarine L
Calcarine R
Cuneus L
Cuneus R
Lingual L
Lingual R
Occipital–Inf L
Occipital–Inf R
Occipital–Mid L
Occipital–Mid R
Occipital–Sup L
Occipital–Sup R
Number of Voxels
39 46 52 58 65 71 78 84 90 97
Peak Frequency (Hz)
11 33 56 79 102 125 148 171 194 217
Signal Change (%)
sess#2
34 41 48 55 62 69 76 83 90 97
Peak Frequency (Hz)
10 33 55 78 101 124 147 170 192 215
Signal Change (%)
(vi)
S07
sess#1
32 37 42 46 51 56 61 65 70 75
Peak Frequency (Hz)
7 15 23 32 40 48 56 64 73 81
Signal Change (%)
sess#2
34 41 48 55 62 69 76 83 90 97
Peak Frequency (Hz)
6 17 28 40 51 62 74 85 96 108
Signal Change (%)

SFig. 7

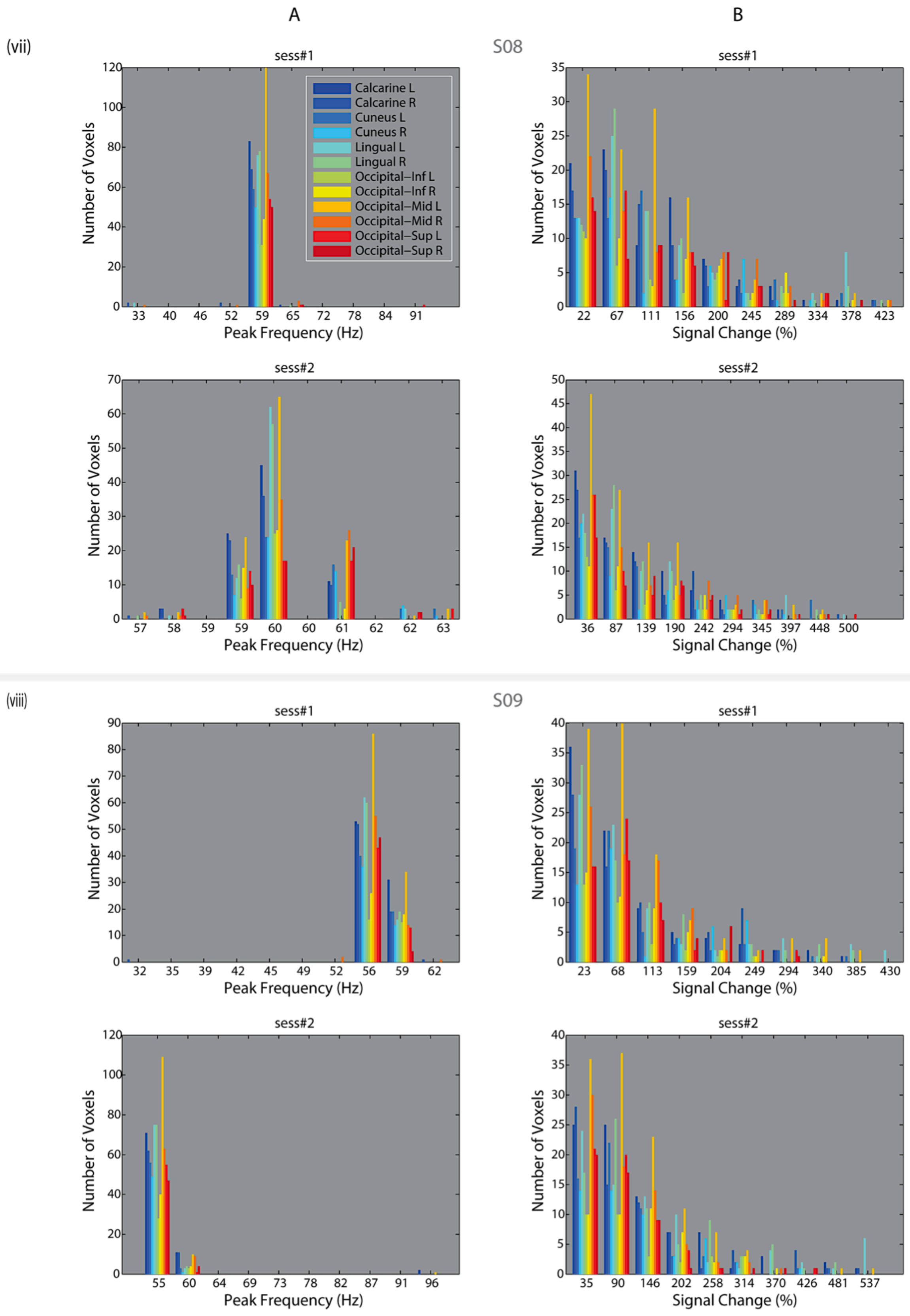
A
B
(vii)
S08
sess#1
Number of Voxels
Peak Frequency (Hz)
33 40 46 52 59 65 72 78 84 91
Calcarine L
Calcarine R
Cuneus L
Cuneus R
Lingual L
Lingual R
Occipital–Inf L
Occipital–Inf R
Occipital–Mid L
Occipital–Mid R
Occipital–Sup L
Occipital–Sup R
Signal Change (%)
22 67 111 156 200 245 289 334 378 423
sess#2
57 58 59 59 60 60 61 62 62 63
36 87 139 190 242 294 345 397 448 500
(viii)
S09
sess#1
32 35 39 42 45 49 52 56 59 62
23 68 113 159 204 249 294 340 385 430
sess#2
55 60 64 69 73 78 82 87 91 96
35 90 146 202 258 314 370 426 481 537

SFig. 7

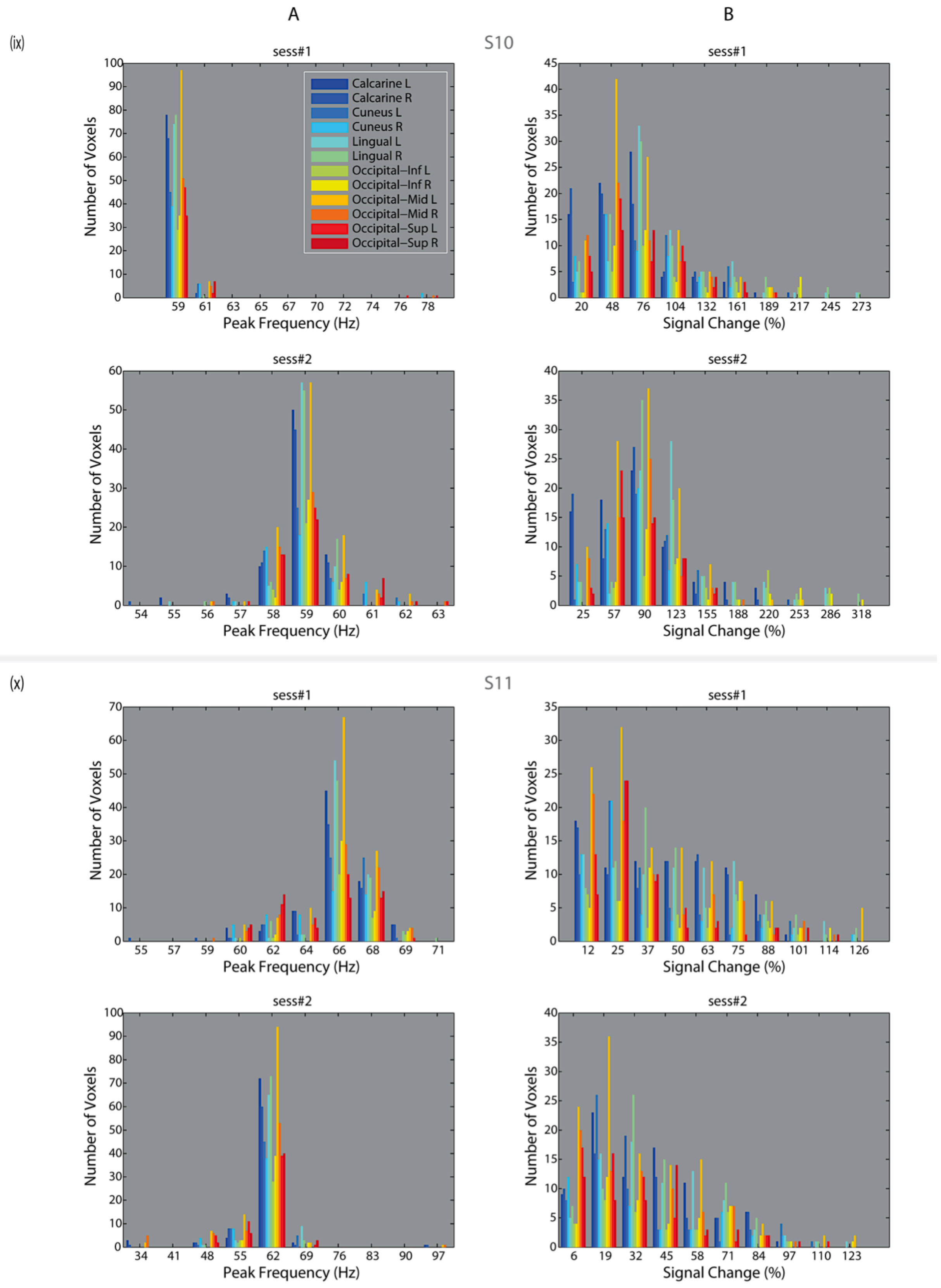

A
B
(ix)
S10
sess#1
Calcarine L
Calcarine R
Cuneus L
Cuneus R
Lingual L
Lingual R
Occipital–Inf L
Occipital–Inf R
Occipital–Mid L
Occipital–Mid R
Occipital–Sup L
Occipital–Sup R
Number of Voxels
Peak Frequency (Hz)
59 61 63 65 67 70 72 74 76 78
Signal Change (%)
20 48 76 104 132 161 189 217 245 273
sess#2
54 55 56 57 58 59 60 61 62 63
25 57 90 123 155 188 220 253 286 318
(x)
S11
sess#1
55 57 59 60 62 64 66 68 69 71
12 25 37 50 63 75 88 101 114 126
sess#2
34 41 48 55 62 69 76 83 90 97
6 19 32 45 58 71 84 97 110 123

SFig. 7

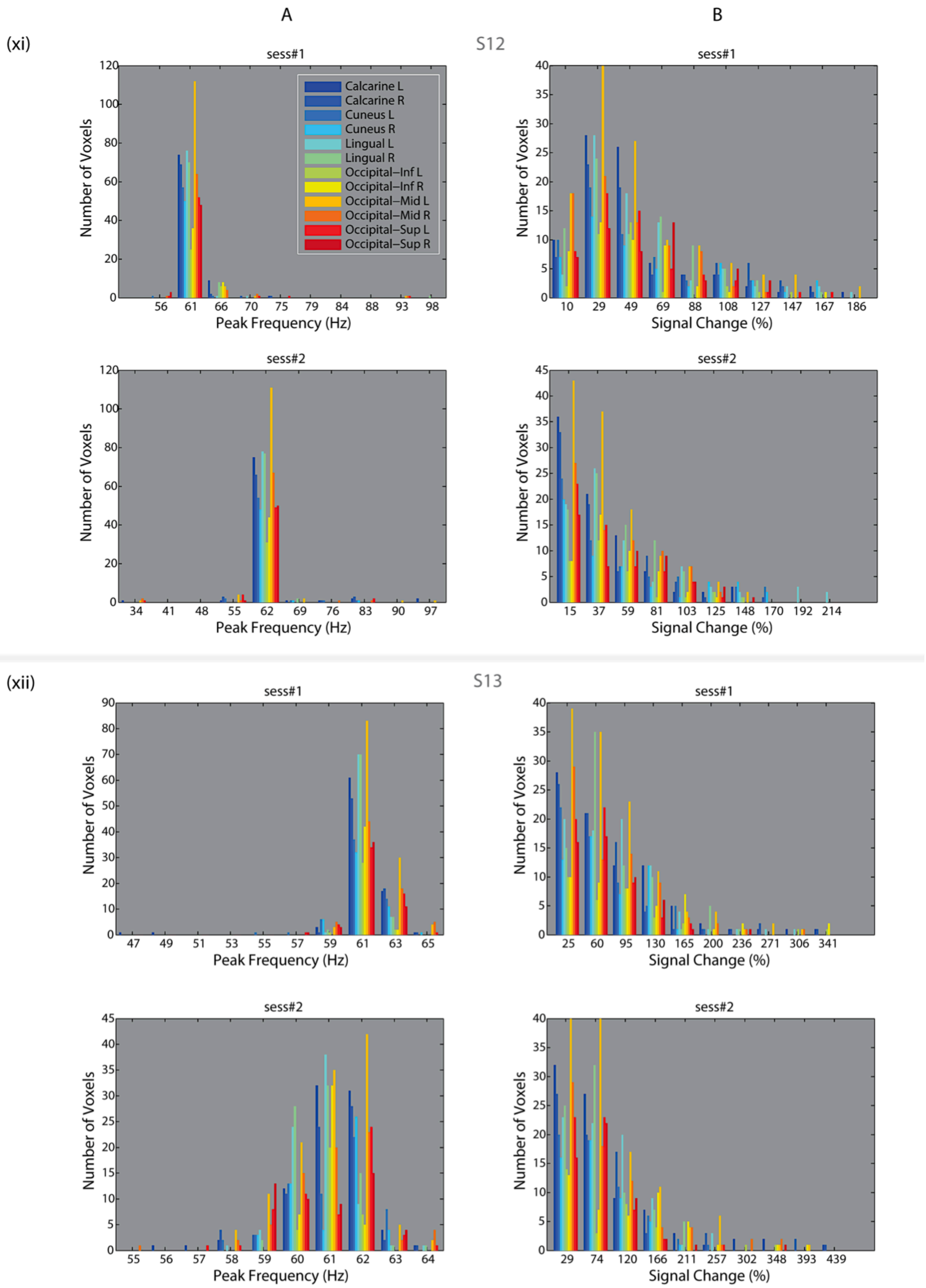

A
B
(xi)
S12
sess#1
sess#2
Calcarine L
Calcarine R
Cuneus L
Cuneus R
Lingual L
Lingual R
Occipital–Inf L
Occipital–Inf R
Occipital–Mid L
Occipital–Mid R
Occipital–Sup L
Occipital–Sup R
Number of Voxels
Peak Frequency (Hz)
Signal Change (%)
(xii)
S13

SFig. 7

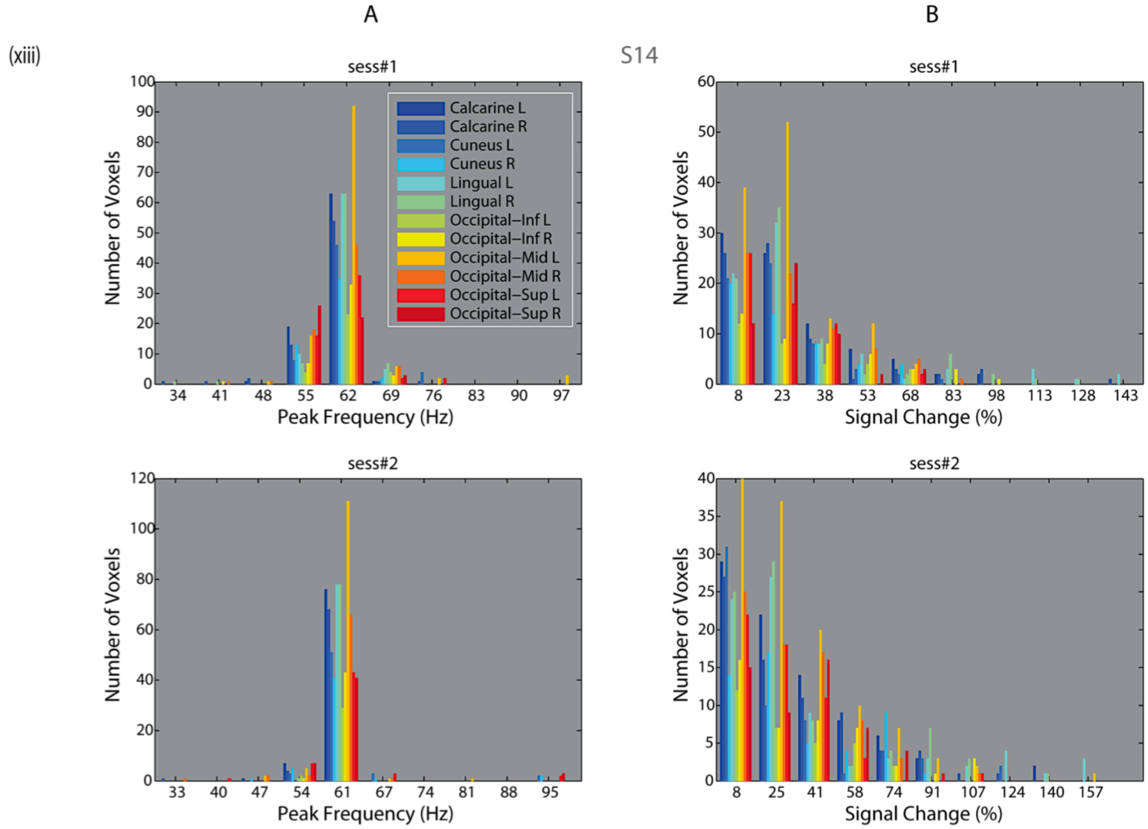
A
B
(xiii)
S14
sess#1
Number of Voxels
Peak Frequency (Hz)
Calcarine L
Calcarine R
Cuneus L
Cuneus R
Lingual L
Lingual R
Occipital–Inf L
Occipital–Inf R
Occipital–Mid L
Occipital–Mid R
Occipital–Sup L
Occipital–Sup R
Signal Change (%)
sess#2

SUPPLEMENTARY FIGURE CAPTIONS:

SFig. 1 Experimental paradigm.

Each trial began with an initial presentation of a central fixation spot (500 ms) whose contrast is subsequently reduced by 40% for a further 1500 ms, prompting the upcoming moving grating presentation. The ensuing concentric moving grating contracted towards the fixation point and accelerated randomly between 750–3000 ms post grating onset. Participants indicated the detection of this acceleration with a button press within 500 ms of its occurrence. Response feedback was provided during the inter-trial interval (1000 ms). Rare incidences (10%) in which no acceleration occurred were interspersed within a series of 80 trials that made up a block of the visual task. Refer to *Methods* for further details.

SFig. 2 Maximally modulated voxels derived for all participants in both experimental sessions.

Each participant's maximally modulated voxels, as determined by non-parametric T-statistics, including their surrounding 26 voxels (Fig. $2_{(iv)}$) are interpolated onto their corresponding MNI-normalized brain volumes. For each session, each participant's maximally modulated voxels in response to moving stimulus grating are shown (from left to right) in coronal, sagittal and axial views. The majority of participants' maximal modulated voxel are located and clustered within the visual cortex. See *Methods* and *Results* for further details.

SFig. 3 Grand average visually induced responses for each MEG session.

Power signal change relative to baseline is averaged across participants separately for signals derived at sensor (i, ii) and at source (iii, iv) for both MEG sessions. Topographies of relative power change for MEG sessions 1 (A) and 2 (B) are shown for (i) all sensors and for (ii) only visual sensors. These signal change topographies are averaged across all participants (i, ii) and aligned on LHS over frequency axes. (ii) Topographies for both the duration of interest 0.5–2 s and frequency range of 50–70 Hz are shown on the RHS above the time-frequency plots. Grand average spectral modulation within visual voxels (iii) and in voxels yielding maximal sensory modulations (iv) is shown. The spectral topography plots (i, ii) highlight that the main contribution for the observed spectral response from all sensors (i) is clustered within the visual-sensors (ii) chosen a priori for the sensor-level analyses. Furthermore, spectral modulation was enhanced in sources and maximally modulated relative to baseline (iv), compared to visual-region-specific source spectral modulations (iii).

SFig. 4 Analysis-approach for deriving visually induced response parameters of interest.

Overview of analysis-approach illustrated with data from 1 participant (S01). For each participant, power signal change relative to baseline was derived at both sensor (i) and source (ii), and for each MEG session (session 1 — upper, and session 2 — bottom rows, respectively). (A) Time-frequency plots for signal change, (B) the corresponding baseline (green) and stimulus-related (red) signals are depicted for both MEG sessions (C). Subsequently, (D) Gaussian fitting of time series was performed for both MEG sessions (solid = average frequency signal change time series; dotted = fitted Gauss for sessions 1 and 2, respectively), from which the parameters: peak frequency, the corresponding signal change and frequency bandwidth were extracted for reliability assessment. Time range for plot illustration is from – 1 to 1.75 s. Refer to *Methods* and *Results* for further details.

SUPPLEMENTARY FIGURE CAPTIONS – continued.

SFig. 5 Distributions of peak frequency and spectral modulation of visual cortical voxels.

Histograms of peak frequency (A) and spectral signal change (B) derived from each individual's (~ 800 voxels within) AAL-parcelled visual cortical areas are shown for both MEG sessions and for all participants: S01, S02, S04–S08 (i) and S09–S14 (ii).

SFig. 6 Individual visual cortical voxel's peak frequency as a function of spectral modulation.

The peak frequency derived for each individual participant's (~ 800) AAL-parcelled visual cortical voxel is shown as a function of corresponding spectral signal change with reference to AAL-parcelled voxel labels (A). The peak frequency and signal change derived from any maximally task-induced cluster of voxels corresponding to the AAL-parcelled visual cortical voxels are overlaid onto each participant's plots (S01, S02, S04–S08 (i) and S09–S14 (ii)) of peak frequency and spectral signal change for MEG sessions 1 (B) and 2 (C). The bins and tick-marks of peak frequency on the x-axis in (B, C) follow the histogram binning in SFig. 5A, which differs from SFig. 6A.

SFig. 7 Distributions of peak frequency and spectral modulation by AAL-parcelled visual regions.

Histograms of peak frequency (A) and spectral signal change (B) derived from each individual's (~ 800 voxels within) AAL-parcelled visual cortical areas are shown for both MEG sessions and for all participants: (i)–(xiii).

http://dx.doi.org/10.1016/j.neuroimage.2016.05.006
